# Trilobatin Protects Against Aβ_25–35_-Induced Hippocampal HT22 Cells Apoptosis Through Mediating ROS/p38/Caspase 3-Dependent Pathway

**DOI:** 10.3389/fphar.2020.00584

**Published:** 2020-05-19

**Authors:** Nana Chen, Jiao Wang, Yuqi He, Yingshu Xu, Yuchuan Zhang, Qihai Gong, Changyin Yu, Jianmei Gao

**Affiliations:** ^1^ Department of Clinical Pharmacotherapeutics, School of Pharmacy, Zunyi Medical University, Zunyi, China; ^2^ Key Laboratory of Basic Pharmacology of Guizhou Province, Zunyi Medical University, Zunyi, China; ^3^ Department of Neurology, the Affiliated Hospital of Zunyi Medical University, Zunyi, China; ^4^ Key Laboratory of Basic Pharmacology of Ministry of Education and Joint International Research Laboratory of Ethnomedicine of Ministry of Education, Zunyi Medical University, Zunyi, China

**Keywords:** Alzheimer’s disease, Aβ_25–35_, HT22 cells, Trilobatin, p38, Sirt3

## Abstract

Emerging evidence reveals that an aberrant accumulation of β-amyloid (Aβ) is the main reason of Alzheimer’s disease (AD) pathogenesis. Thus, inhibition of Aβ-induced neurotoxicity may be promising therapeutic tactics to mitigate AD onset and advance. The development of agent candidates by cultured neurons against Aβ-induced cytotoxicity is widely accepted to be an efficient strategy to explore the drug for AD patients. Previously, we have revealed that trilobatin (TLB), a small molecule monomer, derives from *Lithocarpus polystachyus* Rehd, possessed antioxidative activities on hydrogen peroxide-induced oxidative injury in PC12 cells. The present study was designed to investigate the effects and the underlying mechanism of TLB on Aβ-induced injury in hippocampal HT22 cells. The results demonstrated that TLB attenuated Aβ_25–35_-induced HT22 cell death, as evidenced by MTT assay and LDH release. Furthermore, TLB dramatically mitigated cell death after Aβ_25–35_ insulted *via* decreasing the intracellular and mitochondrial ROS overproduction and restoring antioxidant enzyme activities, as well as suppressing apoptosis. Of note, Aβ_25–35_ triggered increase in ratio of Bax/Bcl-2, activation of caspase-3, phosphorylation of tau, JNK, p38 MAPK, and decrease in Sirt3 expression, whereas TLB reversed these changes. Intriguingly, TLB could directly bind to p38, as evidenced by molecular docking and p38 inhibitor. Taken together, the results reveal that TLB effectively protects against Aβ_25–35_-induced neuronal cell death *via* activating ROS/p38/caspase 3-dependent pathway. Our findings afford evidence for the potential development of TLB to hinder neuronal death during AD.

## Introduction

Alzheimer’s disease (AD), the extremely common type of senile dementia, which is accompanied by progressive learning and memory impairment, as well as cognitive deficits (Deng et al., 2017; Suh et al., 2019). Thus, AD imposes financial, social, and health care burdens around the world. AD is characterized by the aggregation of β-amyloid (Aβ) plaques, loss of neurofibrillary tangles (NFTs) and neurons (Hashimoto et al., 2019). In normal physiological conditions, Aβ does not exhibit any pathological activity. Whereas, it is believed that Aβ deposition is the major element of senile plaques discovered in the brains of AD patients (Weiner et al., 2017). Notably, the aberrant accumulation of Aβ in neurons involving complex detrimental mechanisms, including oxidative stress injury, mitochondrial dysfunction, which further results in neuronal loss, even death (Cioffi et al., 2019). Therefore, inhibition of Aβ-induced neurotoxicity may be a promising therapeutic strategy to mitigate AD onset and advance. Nevertheless, to date, desired approaches or drugs are still unavailable due to the low efficacy or severe adverse drug reaction such as acetyl cholinesterase inhibitors or antagonists of *N*-methyl-d-aspartate receptor (Arvanitakis et al., 2019). Hence, the exploit of safe and efficient treatment strategies for AD are still an urgent clinical need. It is worth noting that a lot of neuroprotectants for AD failed in clinical trials, mainly due to an encounter with the hardness in affording therapeutic proteins or drugs to the central nervous system (Yuan et al., 2019). Therefore, it is reasonably assumed that small molecule monomer, primarily derive from the natural product, will be an alternative to affect neurons.

Trilobatin (TLB) is a glycosylated dihydrochalcone compound isolated from a traditional Chinese folk medicine *Lithocarpus polystachyus* Rehd (Wang et al., 2016).

TLB was not only used as a natural sweetener, but also exerted multiple pharmacological activities, including, antioxidative properties (Yang et al., 2004; Li et al., 2018), anti-diabetes mellitus (Wang et al., 2016), anti-inflammation (Fan et al., 2015), anti-hepatoblastoma cells proliferation as a novel SGLT1/2 inhibitor (Wang L et al., 2019), anti-HIV-1 activity (Yin S. et al., 2018). Intriguingly, our previous study has revealed that TLB significantly protected against hydrogen peroxide (H_2_O_2_)-induced oxidative injury in a neuron-like PC12 cell (Gao et al., 2018). Moreover, since oxidative injury plays a vital role during neuronal damage induced by Aβ (Zhou et al., 2016), it is assumed that TLB could also mitigate Aβ-induced toxicity in neurons. Thus, based on the above scenario, the current study was designed to evaluate the hypothesis whether TLB could protect against Aβ_25–35_-induced injury in the murine hippocampal neuron HT22 cells, and further to explore its detailed mechanism.

## Materials and Methods

The murine hippocampal neuronal cell line HT22 cells were from American Type Culture Collection (Rockville, MD, USA). TLB (purity ≥ 98% by HPLC) was purchased from Nanjing Zelang Medical Technology Corporation Ltd (Nanjing, China). TLB was dissolved in dimethyl sulfoxide (DMSO) at 10 mM as stock solution and the final concentration of DMSO in the media was less than 0.1%. 3-(4,5-dimethylthiazol-2-yl)-2,5-diphenyltetrazolium bromide (MTT, #M2128), Aβ_25–35_(#A4559), and 2′,7′-dichlorodihydrofluorescein diacetate (DCFH-DA, #D6883), Z-Aspartic acid-Glutamic acid-Valine-Aspartic acid-fmk (Z-VAD-FMK, #V116), SB203580 (#S8307) were from Sigma-Aldrich (St Louis, MO, USA). Fetal bovine serum (FBS, #10099141) and Dulbecco’s modified Eagle’s medium (DMEM,# 12430047) were from Gibco BRL (Gaithersburg, MD, USA). Mito-SOX Red (#M36008) was purchased from Invitrogen (Eugene, OR, USA). One step *in situ* cell death detection kit (#RJ2030), mouse lactate dehydrogenase (LDH, #RJ17549) ELISA kit, malondialdehyde (MDA, #RJ16984), superoxide dismutase (SOD, #RJ17004) glutathione peroxidase (GSH-Px, #RJ17154), and glutathione (GSH, #RJ RJ14558) ELISA kits were from Shanghai Renjie Biotechnolgy (Shanghai, China). Caspase-Glo ^®^ 3/7 Assay kit (#G8091) was obtained from Promega (Promega, USA). The antibodies against α-tubulin and GAPDH were from TransGen Biotech (Beijing, China). The antibodies against tau (#ab32057), phospho-tau (#ab109390), Bax (#ab32503), Bcl-2 (#ab59348), caspase-3 (#ab13847), cleaved caspase-3(#ab49822), p38 (#ab170099), phospho-p38 (#ab4822), c-Jun N-terminal kinase (JNK, # 179461), phospho-JNK(#ab124956), and Sirt3 (#ab118334) were from Abcam (Cambridge, UK).

### Cell Culture and Drug Treatment

The cells were cultured in DMEM containing 10% FBS and 1% penicillin/streptomycin at 37°C in a humidity chamber with 5% (v/v) CO_2_. Aβ_25–35_ was diluted to 1 mM with sterilized saline water and then incubated at 37°C for 7 days before use (Ghasemi et al., 2014). Thereafter, the HT22 cells were treated with or without 12.5, 25, and 50 μmol/L TLB or 10 μmol/L Z-VAD-FMK (a caspase-3 inhibitor), or 20 μmol/L SB203580 (a p38 inhibitor) and 20 μmol/L Aβ_25–35_ for 48 h.

### MTT Assay

At the end of treatment as described above. The HT22cells were cultured with MTT (5 mg/ml) for another 4 h, then, the medium was carefully removed, and 150 μl DMSO was added into each well. Thereafter, the optical density value of the cells was measured by a microplate reader at a wavelength of 490 nm. Cell viability was expressed as the percentage relative to the absorbance of the untreated control cells.

### LDH Release Assay

Neurotoxicity was determined by the release of LDH, an indicator of cytotoxicity. In brief, the HT22 cells were treated as described above, then the supernatant were collected by centrifugation (2,000*g*, 20 min), and the LDH release was evaluated using an LDH assay kit according to the protocol instruction. Thereafter, absorbance was detected at wavelength of 490 nm, and all values of % LDH released were normalized to the untreated control group. Additionally, changes in cellular morphology were simultaneously observed by phase contrast microscopy.

### TUNEL Assay

The apoptosis of HT22 cells was detected using the TUNEL assay by the *In Situ* Cell Death Detection Kit according to the manufacturer’s instructions. Briefly, the HT22 cells were treated as described above, then the cells were fixed with 4% paraformaldehyde for 30 min at room temperature, and then incubated with 0.3% Triton X-100 in PBS for 10 min. Thereafter, the HT22 cells were incubated in TUNEL test solution at 37°C for 1 h in the dark, while, the cell nuclei were labeled using DAPI staining. Thereafter, TUNEL-positive cells were observed using a fluorescence microscope (Olympus IX73, Olympus, Tokyo, Japan) with excitation/emission (450/515 nm) wavelengths.

### Measurement of Caspase 3/7 Activity

The HT22 cells were treated as described above, and the Caspase-Glo ^®^ 3/7 Assay was used to evaluate the caspase 3/7 activity according to the manufacturer’s protocols. The cells were lysed in lysis buffer, and the supernatants were collected with centrifugation (12,500 × g for 5 min at 4°C), then the supernatants were co-incubated with working solution in 96-well plates for 30 min at room temperature. Thereafter, multimode reader with excitation/emission (490 nm/520 nm) wavelengths was used to detect the fluorescence intensity. All values of caspase 3/7 activities were expressed as the percentage relative to the absorbance of the untreated control cells.

### Measurement of Intracellular ROS and Mitochondrial ROS (mtROS)

The HT22 cells were treated as described above. Intracellular ROS generation was evaluated by DCFH-DA fluorescence probe. Briefly, the cells were loaded with 20 μM DCFH-DA for 30 min in the dark, thereafter, washed twice with PBS and then the fluorescence was observed and imaged under a fluorescent microscope (Olympus IX73; Olympus, Tokyo, Japan) with excitation/emission (485/530 nm) wavelengths. Image Pro-Plus software was used to calculate intracellular ROS, and all values of ROS level were expressed as a percentage compared with the control. Furthermore, **t**he Mito-SOX Red fluorescent probe, a fluorescent indicator for mitochondrial O_2_^•^^−^, was used to evaluate mtROS. In brief, the cells were incubated in 5 μM Mito-SOX Red fluorescent probe at 37°C in dark for 30 min, while, the cell nuclei were labeled using DAPI staining. Afterwards, the cells were imaged by fluorescence microscopy (Olympus IX73; Olympus, Tokyo, Japan) with excitation/emission (510/580 nm) wavelengths.

### Measurement of MDA

The level of MDA, a product of lipid peroxidation, was measured using MDA ELISA kit. In brief, the HT22 cells were treated as described above, then culture supernatant was collected by 0.25% trypsin, centrifuge at 3000 × g for 3 min. The level of MDA in the supernatant was detected according to the manufacturer’s recommendations.

### Measurement of Antioxidant Enzyme Activities

Evaluation of antioxidantive enzyme activities were measured using SOD, GSH-Px, and GSH assay kits. Briefly, the HT22 cells were treated as described above, then the supernatants were collected with centrifugation (3000 × g for 10 min at 4°C). Thereafter, the activities of SOD, GSH-Px, and GSH content were determined according to the manufacturer’s instructions.

### Western Blot Analysis

Briefly, the HT22 cells were treated as described above, then protein concentration was evaluated by BCA assay, and lysates were normalized to equal amounts of protein. Thereafter, 20 μg protein from cell lysates were separated by SDS-PAGE (10% - 12%) and transferred onto a PVDF membrane. Then membranes were blocked with 5% nonfat milk in TBST for 1 h at room temperature. and then incubated overnight with appropriate primary antibodies: anti-p-tau, anti-tau, anti-p-JNK, anti-JNK, anti-p-p38, anti-p38, anti-Sirt 3 (1:1000), anti-Bcl-2 (1:1000), anti-Bax (1:1000), anti-cleaved-caspase-3 (1:1000), anti-caspase-3 (1:1000), α-tubulin (1:2000), and anti-GAPDH (1:2000) at 4°C. Then the membranes were incubated with HRP-conjugated secondary antibody (1:5000) at room temperature for 2 h. Then, representative bands were visualized by ECL according to the manufacturer’s instructions, and Image J software was used to quantify the band optical intensity.

### Quantitative Real-Time PCR (qRT-PCR)

Total RNA was extracted with the Trizol Reagent, which was reverse transcribed to cDNA with the PrimeScript™ RT Reagent Kit. qRT-PCR was performed on the CFX96 real-time PCR detection system (Bio-Rad Laboratories Ltd, Hertfordshire, UK) with specific primers, and their sequences were listed as follows: mouse p38e, forward 5′-TGTGAACGAAGACTGTGAGC-3′ and reverse 5′-GCATCCAATTCAGCATGATCTC-3′; mouse p38e, forward 5′-CGCCAGAAGGTGGCTGTAAA-3′ and reverse 5′-TGTCCTCCTCGCGTGGAT-3′; mouse GAPDH forward 5′-ACAACTTTGGCATTGTGGAA-3′ and reverse 5′-GATGCAGGGATGATGTTCTG-3′. In the reaction, 1 μl cDNA of each sample was mixed with SYBR^®^GREEN PCR Master Mix according to the protocol of manufacture. And the PCR conditions: 30 s at 95°C, 40 cycles at 95°C for 5 s, followed by 56°C for 60 s. Relative gene expression was evaluated by the 2^−ΔΔCT^ method. The mRNA levels of p38α and p38β in WT neurons were normalized to 1.0.

### Molecular Docking

The affinity between TLB and p38α, p38β, and JNK were performed by Autodock 4.2 and AutodockTools (ADT). In brief, the PDB format file for the p38α (Protein Data Bank ID: 1ZZL), p38β (Protein Data Bank ID: 3GP0), and JNK (Protein Data Bank ID: 4IZY) protein were obtained from the Protein Data Bank database. A three-dimensional structure of TLB was established using the ChemBio3D Ultra 14.0 (PerkinElmer Informatics, USA), which was further proceeded using ADT. Thereafter, the molecular docking of the TLB and p38 proteins was performed using Autodock 4.2. The area of grid box contained the promising binding sites between TLB and JNK or p38 as previous study report (Ma et al., 2018).

### Statistical Analysis

All data were expressed as mean ± standard deviation (SD). A one-way analysis of variance followed by Bonferroni’s test was carried out to test for any differences between individual differences. A *P* value < 0.05 was considered statistically significant. All experiments were performed in triplicate.

## Results

### TLB Concentration-Dependently Inhibited Aβ_25–35_-Induced Cytotoxicity in HT22 Cells

**Figure 1:**
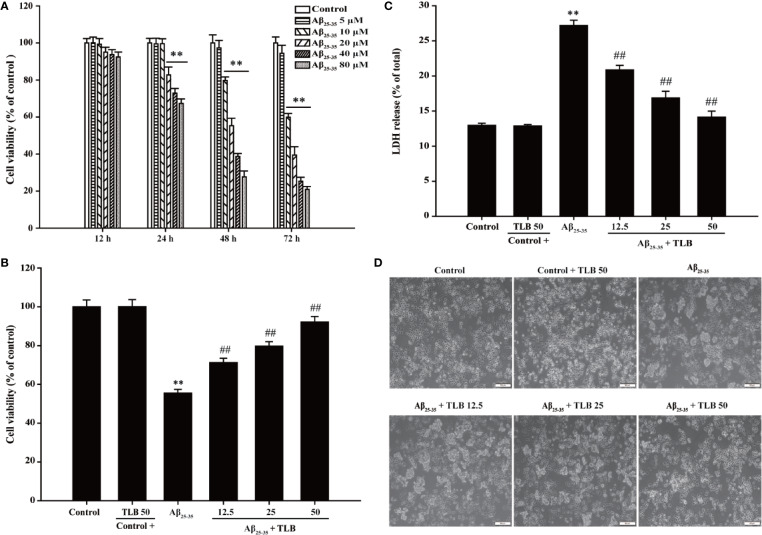
TLB protected against Aβ_25–35_-induced cytotoxicity in hippocampal HT22 cells. **(A)** HT22 cells were exposed to the different concentrations of Aβ_25–35_ (5, 10, 20, 40, and 80 μM) for 12 h, 24 h, 48 h, and 72 h, respectively. The cell viability was determined by MTT assay. HT22 cells were exposed to 20 μM Aβ_25–35_ for 48 h in the presence or absence of TLB (12.5, 25, and 50 μM). **(B)** The cell viability was determined by MTT assay. **(C)** LDH release from HT22 cells was measured using an LDH assay kit. **(D)** The protective effects of TLB on Aβ_25–35_-induced morphological alternation. Data were presented as mean ± SD. (n = 6). ^**^ *P* < 0.01 *versus* control group; ^##^ *P* < 0.01 *versus* Aβ_25–35_ group

The effect of Aβ_25–35_ on HT22 cells were determined, and it was found Aβ_25–35_ decreased cell viability and increased LDH release in a dose- and time-dependent manner. Since 48 h of Aβ_25–35_ (20 μM) incubation apparently induced cytotoxicity to about 50% (**Figure 1A**), the concentration and incubation period were applied for the following experiments. Thereafter, the protective effects of TLB on Aβ_25–35_-induced injury in HT22 cells were determined. The results showed that TLB significantly increased cell viability and decreased LDH release than those of Aβ_25–35_ group, as evidenced by MTT and LDH release assay, respectively (**Figures 1B, C**). Moreover, the beneficial effect of TLB was also verified by morphologic observations. The results revealed that Aβ_25–35_-treated cells led to the decrease in the number of cells and floatation than those of the control group; while, TLB reversed these change than those of Aβ_25–35_ group (**Figure 1D**).

### TLB Suppressed Aβ_25–35_-induced ROS Generation and MDA Content in HT22 Cells

**Figure 2:**
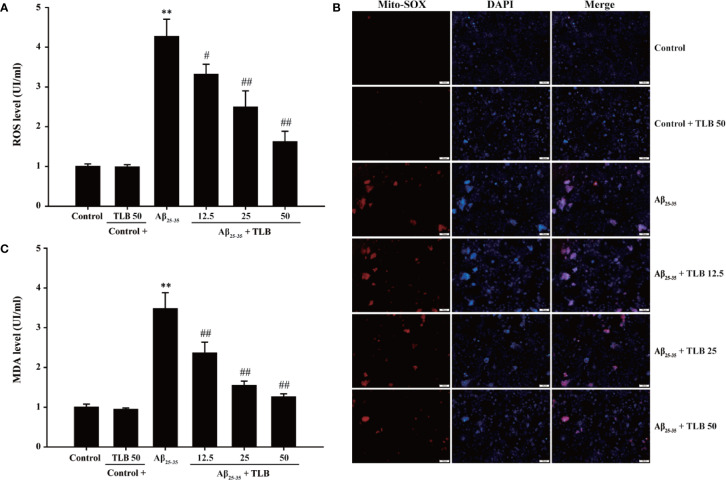
TLB attenuated Aβ_25–35_-induced overproduction of intracellular ROS and mitochondrial ROS (mtROS) and MDA level. HT22 cells were exposed to 20 μM Aβ_25–35_ for 48 h in the presence or absence of TLB (12.5, 25, and 50 μM). **(A)** The intracellular ROS generation was evaluated by DCFH-DA fluorescence probe. **(B)** The Mito-SOX Red fluorescent probe was used to detect mtROS. **(C)** The level of MDA was measured using MDA ELISA kit. Data were presented as mean ± SD. (n = 6). ^**^ *P* < 0. 01 *versus* control group; ^#^ *P* < 0.05, ^##^ *P* < 0.01 *versus* Aβ_25–35_ group.

To investigate whether TLB could suppress Aβ_25–35_-induced oxidative injury in HT22 cells, the generation of intracellular ROS, mtROS, MDA was detected. The results showed that the generation of intracellular ROS, mtROS, as well as MDA was significantly increased after Aβ_25–35_ insulted, consisting with the previous study (Angeloni et al., 2019). Whereas, TLB markedly reversed these change of ROS generation both in intracellular (**Figure 2A**) and mitochondria (**Figure 2B**), as well as MDA content (**Figure 2C**) as evidenced by DCFH-DA staining, Mito-SOX staining, and MDA ELISA kit, respectively. These findings suggested that TLB effectively suppressed Aβ_25–35_-induced oxidative injury in HT22 cells.

### TLB Increased Antioxidant Enzyme Activities in Aβ_25–35_-Induced Injury in HT22 Cells

**Figure 3:**
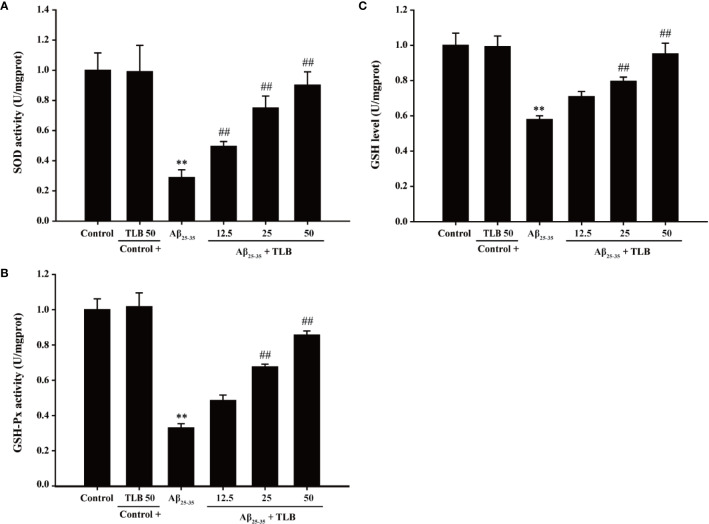
TLB increased antioxidant enzyme activities in Aβ_25–35_-induced injury in HT22 cells. HT22 cells were exposed to 20 μM Aβ_25–35_ for 48 h in the presence or absence of TLB (12.5, 25, and 50 μM). **(A)** The activities of SOD were measured using SOD ELISA kit. **(B)** The activities of GSH-Px were measured using GSH-Px ELISA kit. **(C)** The content of GSH was determined by GSH ELISA kit. Data were presented as mean ± SD. (n = 6). ^**^ *P* < 0.01 *versus* control group; ^##^ *P* < 0.01 *versus* Aβ_25–35_ group.

To evaluate the effect of TLB on antioxidant enzyme activities in HT22 cells after Aβ_25–35_ insulted, the activities of SOD, GSH-Px, and the content of GSH were determined. The results showed that Aβ_25–35_ significantly decreased SOD and GSH-Px activities, and GSH content, in keeping with the previous study (Campolo et al., 2018). Whereas, TLB significantly reversed these change of SOD (**Figure 3A**) and GSH-Px (**Figure 3B**), as well as GSH content (**Figure 3C**), which indicated that TLB effectively promoted antioxidant enzyme activities in Aβ_25–35_-induced injury in HT22 cells.

### TLB Mitigated Aβ_25–35_-Induced Injury in HT22 Cells Through Mediating Caspase-3-Dependent Apoptosis Pathway

**Figure 4:**
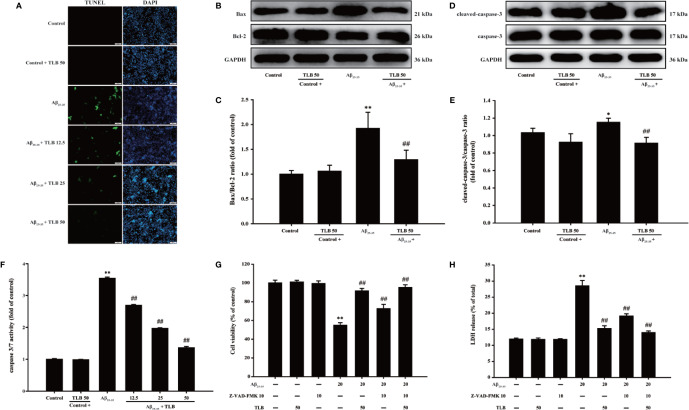
TLB mitigated Aβ_25–35_-induced apoptosis in hippocampal HT22 cells. HT22 cells were exposed to 20 μM Aβ_25–35_ for 48 h in the presence or absence of TLB (12.5, 25, and 50 μM) or Z-VAD-FMK (10 μM), a caspase-3 inhibitor. **(A)** Apoptosis cells determined with TUNEL staining (green). **(B)** Representative Western blot images of Bax and Bcl-2. **(C)** Quantitation of Bax/Bcl-2 ratio. (n = 5) **(D)** Representative Western blot images of cleaved-caspase-3 and caspase-3. **(E)** Quantitation of cleaved-caspase-3. (n = 5). **(F)** The caspase 3/7 activity was evaluated by the Caspase-Glo®3/7 assay. (n = 5). HT22 cells were pre-treated with Z-VAD-FMK, a pan-caspase inhibitor, prior 1 h to TLB treatment. **(G)** The cell viability was determined by MTT assay. **(H)** LDH release from HT22 cells was measured using an LDH assay kit. Data are presented as mean ± SD. (n = 6). ^*^ *P* < 0. 05; ^**^ *P* < 0.01 *versus* control group; ^##^ *P* < 0.01 *versus* Aβ_25–35_ group.

The effect of TLB on Aβ_25–35_-induced apoptosis in HT22 cells were detected using TUNEL staining. Aβ_25–35_ triggered apoptosis in HT22 cells as evidenced by the increasing amounts of TUNEL-positive cells, and this apoptotic effect was significantly attenuated by TLB, which suggested that TLB could inhibit Aβ_25–35_-induced apoptosis in HT22 cells (**Figure 4A**). Furthermore, Bax/Bcl-2 ratio, cleaved-3 level, and caspase-3 activity were increased upon Aβ_25–35_, whereas TLB significantly reversed these change of Bax/Bcl-2 ratio (**Figures 4B, C**), cleaved-caspase-3 level (**Figures 4D, E**), and caspase-3 activity (**Figure 4F**) in HT22 cell injury induced by Aβ_25–35._ Intriguingly, to further confirmed the role of caspase activation involved in the attenuation effect of TLB on Aβ_25–35_-induced apoptosis, HT22 cells were pre-treated with Z-VAD-FMK, a pan-caspase inhibitor, prior 1 h to TLB treatment. The results revealed that Z-VAD-FMK significantly abolished the cytotoxicity induced by Aβ_25–35_ in HT22 cells, as evidenced by MTT assay and LDH release (**Figures 4G, H**). Collectively, these findings indicated that TLB suppressed Aβ_25–35_-induced injury in HT22 cells, at least partially, *via* mediating caspase- 3-dependent apoptosis pathway.

### p38/Sirt3 Pathway Mediated the Inhibitory Effects of TLB on HT22 Cells Injury Induced by Aβ_25–35_

**Figure 5:**
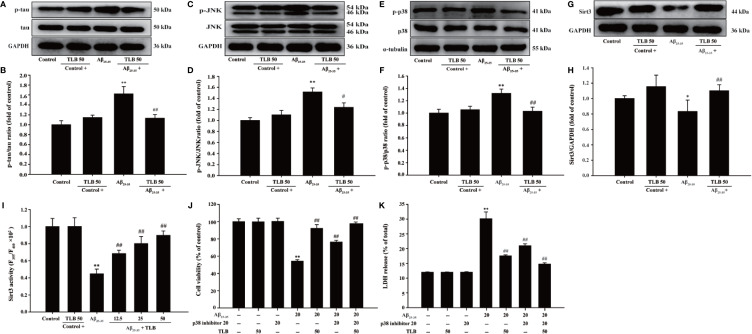
The effects of TLB on Aβ_25–35_-induced hippocampal HT22 cells oxidative injury. **(A)** Representative Western blot images of p-tau and tau. **(B)** Quantitation of p-tau. (n = 5). **(C)** Representative Western blot images of p-JNK and JNK. **(D)** Quantitation of p-JNK. (n = 5). **(E)** Representative Western blot images of p-p38, p38. **(F)** Quantitation of p-p38. (n = 5). **(G)** Representative Western blot images of Sirt3. **(H)** Quantitation of Sirt3. (n = 5). **(I)** The Sirt3 activity was measured using Sirt3 activity assay kit. (n = 6). HT22 cells were pre-treated with SB203580, a p38 inhibitor, prior 1 h to TLB treatment. **(J)** The cell viability was determined by MTT assay. **(K)** LDH release from HT22 cells was measured using an LDH assay kit. Data are presented as mean ± SD. ^*^ *P* < 0.05; ^**^ *P* < 0.01 *versus* control group; ^##^ *P* < 0.01 *versus* Aβ_25–35_ group.

The detailed neuroprotective mechanism of TLB on Aβ_25–35_-induced HT22 cell injury was explored. The results showed that the phosphorylation of tau, JNK and p38 was significantly increased, while the expression and activity of Sirt3 were markedly decreased after Aβ_25–35_ insulted. However, TLB effectively decreased the phosphorylation of tau (**Figures 5A, B**), JNK (**Figures 5C, D**), and p38 (**Figures 5E, F**) and increased both the Sirt3 expression (**Figures 5G, H**) and its activity (**Figure 5I**). Furthermore, the neuroprotective effect of TLB on Aβ_25–35_-induced HT22 cell injury was significantly promoted by p38 inhibitor SB203580. The results revealed that SB203580 significantly abolished the cytotoxicity induced by Aβ_25–35_ in HT22 cells, and promoted the beneficial effect of TLB, as evidenced by MTT assay and LDH release (**Figures 5J, K**). These findings suggested that the role of p38/Sirt3 pathway was involved in the inhibitory effects of TLB on HT22 cells injury induced by Aβ_25–35_.

### Prediction of Drug Targets of TLB Against Aβ_25–35_-Induced Injury in HT22 Cells

**Figure 6:**
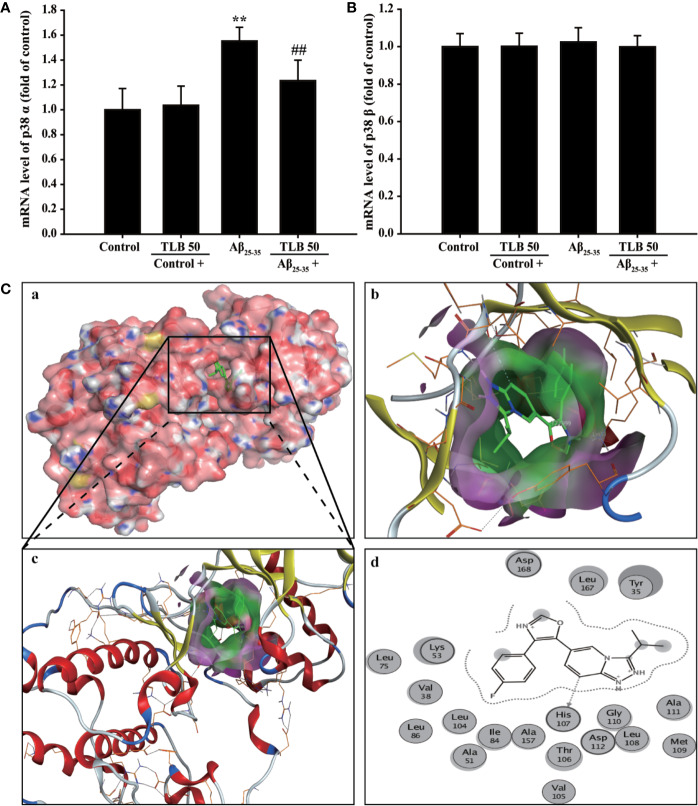
Prediction of drug targets of TLB against Aβ2_5–35_-induced injury in HT22 cells. **(A)** p38α mRNA level. (n = 5). **(B)** p38β mRNA level. (n = 5). **(C)** The binding energy has been surfaced and key residues of TLB with p38 were displayed using in silico molecular docking. (a) The substrate binding surface. (b) The substrate binding sites. (c) The substrate binding pocket. (d) Amino acid residues. Data are presented as mean ± SD. ^**^ *P* < 0.01 *versus* control group; ^##^ *P* < 0.01 *versus* Aβ_25–35_ group.

To further dissect which isoform of p38 is involved in the beneficial effect of TLB on Aβ_25–35_-induced injury, the p38α and p38β mRNA levels were determined using qRT-PCR. The results showed that Aβ_25–35_ significantly increased mRNA level of p38α isoform, but not affect p38β isoform; whereas, TLB effectively decreased mRNA level of p38α, but not affect p38β (**Figures 6A, B**). Furthermore, the molecular docking was used to explore the potential target of TLB during the Aβ_25–35_-induced HT22 cell injury. Interestingly, it was found that the binding energy of TLB with JNK, p38α and p38β was −4.21, −6.05, and −4.45 kcal/mol, respectively. The suppositive binding modes and interaction within the amino acid pocket of TLB with p38α, including LYS53, HIS107, GLY110, ALA111, TYR35, and LEU167, which further confirmed that TLB bound to the hydrophobic pocket of p38α, in keeping with the results *in vitro* (**Figure 6C**). These findings indicated that the TLB might directly bound to p38α to develop its pharmacological activities.

## Discussion

**Figure 7:**
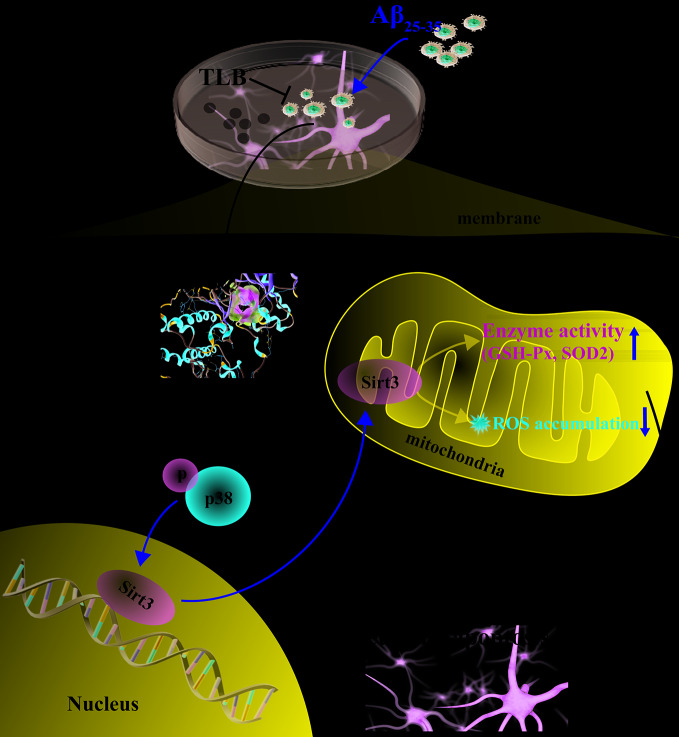
Presentation of a proposed mechanism for the neuroprotective role of TLB against Aβ_25–35_-induced oxidative injury in hippocampal HT22 cells. TLB is thought to direct bind p38 and inhibit p38 phosphorylation, thereby increasing Sirt3 expression and then reducing overproduction of ROS both in intracellular and mitochondria, and elevating antioxidant enzyme activities. The beneficial effects of TLB on Aβ_25–35_-induced neuronal apoptosis, at least partly, through mediating ROS/p38/caspase 3-dependent pathway.

The current study disclosed that: (1) TLB, a naturally-small molecule monomer, derived from herbal *Lithocarpus polystachyus* Rehd. protected against Aβ_25–35_-induced injury in HT22 cells; (2) The beneficial effects of TLB were due to suppression of caspase-3-dependent apoptosis pathway, as well as inhibition of oxidative injury *via* mediating p38/Sirt3 pathway; (3) p38α might be the potential target of TLB as evidenced by molecular docking (**Figure 7**).

Emerging evidence suggests that the extracellular deposition of Aβ plaques and the accumulation of intracellular NFTs are deemed to be the most vital pathophysiology of AD. Aβ exposure could exacerbate tau-mediated neurotoxicity *via* facilitating tau phosphorylation (Holubova et al., 2019). In the present study, we found that Aβ_25–35_ apparently injured HT22 cells and promoted tau protein phosphorylation, consistenting with previous report(Lee et al., 2016). TLB not only effectively attenuated Aβ_25–35_-induced injury in HT22 cells, but also inhibited tau protein phosphorylation, which suggested that TLB could suppress Aβ-induced neurotoxicity by restraining tau phosphorylation.

Recent studies reveal that oxidative stress injury plays an important role in pathogenesis of AD with the markers of ROS and MDA overproduction and antioxidant enzyme activities reduction(Dong et al., 2018; Ji et al., 2019). The results showed that Aβ_25–35_ significantly induced ROS overproduction overwhelmed antioxidant enzyme system, leading to cellular redox balance disequilibrium. Whereas TLB dramatically abolished Aβ_25–35_-induced oxidative stress injury through reducing ROS overproduction and augmenting antioxidant enzyme activities, which suggested that the beneficial effect of TLB on Aβ_25–35_-induced hippocampal neuron injury was attributed to its antioxidant properties. Furthermore, Bcl-2 and Bax are the vital members of Bcl-2 family proteins, and these proteins mediate multiple effectors thereby activating caspases in apoptosis(Sun et al., 2016). Of note, caspases, especially caspase-3, an eventual protein in the apoptotic cascade, which catalyzes the specific cleavage of multiple vital cellular proteins(Zhang et al., 2019). In this study, Aβ_25–35_-induced hippocampal neuron apoptosis by facilitating upregulationg of Bax/Bcl-2 ratio and cleaved-caspase-3 level. While TLB effectively reversed these change, suggesting that TLB mitigated Aβ_25–35_-induced oxidative injury in HT22 cells, at least partly, through mediating caspase-3-dependent apoptosis pathway.

Notably, the silent information regulator 2 (Sir2) family is mechanically crucial in neurons upon oxidative stress(Liu et al., 2018). Sirtuin3 (Sirt3) is a vital deacetylase among the seven subtypes of SIR2 family, which has ability of scavenging ROS and elevating antioxidant enzyme activities (Qin et al., 2018). Interestingly, a recent study has shown that Sirt3 is abundant in the brain, and it might be a mediator that connects Aβ (Yin J. et al., 2018). Thus, we focus on the role of Sirt3 during the beneficial effect of TLB on HT22 cell injury challenged by Aβ_25–35_. Our findings demonstrated that Aβ_25–35_ led to Sirt3 expression and activity decrease, in keeping with the previous studies (Yin J. et al., 2018; Liu et al., 2019). Whereas TLB reversed the decline in Sirt3 expression and activity after Aβ_25–35_ insults, which indicated that Sirt3 contributed to the attenuation effects of TLB on Aβ_25–35_-induced oxidative injury in hippocampal neuron. It is worth noting that Sirt3 will be downregulated *via* activating mitogen-activated protein kinase (MAPK) (Shen et al., 2019), which is a family of serine-threonine kinases and has been considered to play a crucial role in the regulation of oxidative stress and cell death (Kim and Sieburth, 2018). MAPK signaling pathways are related to the pathogenesis of a variety of neurodegenerative diseases, such as AD (Chang et al., 2018). Upon exposure to Aβ stimuli, JNK and p38 are activated in neurons (Wang Q. et al., 2019). Our findings found that phosphorylation of JNK and p38 showed a remarkable increment after Aβ insults; whereas, TLB significantly reversed these change. Furthermore, the inhibitory effect of TLB on Aβ_25–35_-induced hippocampal neuron injury was substantially promoted by p38 inhibitor. Of note, there are four major isoforms of p38 (α, β, γ, and δ), which are expressed in different tissues and cell types and often functionally distinct (Bachstetter and Van Eldik, 2010). Among them, p38α is expressed in most tissues and cell types, p38β is in brain, p38γ in endocrine glands, and p38δ in skeletal muscle. Therefore, in attempting to elucidate the role of p38 in the suppressive effect of TLB on Aβ_25–35_-induced neuronal injury, it is necessary to dissect between p38α and p38β. The results further indicated that p38α mRNA level was increased, but not affected p38β after Aβ insulted, consisting with previous report (Schnoder et al., 2016). TLB mitigated the mRNA level of p38α, but not affect p38β, suggesting that TLB selectively inhibition of p38α to attenuated Aβ-induced hippocampal neuron injury. Intriguingly, TLB could directly bind to p38α, but did not bind to p38β, as well as JNK, as evidenced by molecular docking. Thus, it is reasonable to speculate that p38α might be the potential target of TLB. These findings suggested that TLB effectively protected against Aβ_25–35_-induced injury in hippocampal neuron, at least partly, through inhibiting the MAPK pathway and then activated Sirt3.

## Conclusion

In summary, this study highlights a crucial role of the ROS/p38/caspase 3-dependent pathway in the attenuation effect of TLB on Aβ_25–35_-induced apoptosis in hippocampal neurons. Our findings provide evidence for the potential development of TLB to hinder neuronal death during AD, thereof the neuroprotective effect of TLB on AD is priority to further explore *in vivo*.

## Data Availability Statement

The original contributions presented in the study are included in the article/**Supplementary Material**, further inquiries can be directed to the corresponding authors..

## Author Contributions

JG and QG conceived and designed the experiments, NC, JW, and YZ performed the experiments. YX and YH analyzed the data. NC wrote the manuscript. CY and JG revised the manuscript. All authors read and commented on the manuscript.

## Conflict of Interest

The authors declare that the research was conducted in the absence of any commercial or financial relationships that could be construed as a potential conflict of interest.
