# Supplementary figures and images for: Trilobatin Protects Against Aβ25–35-Induced Hippocampal HT22 Cells Apoptosis Through Mediating ROS/p38/Caspase 3-Dependent Pathway
Source: Front Pharmacol. 2020 May 19;11:584. doi: 10.3389/fphar.2020.00584 (PMC7248209; doi:10.3389/fphar.2020.00584)

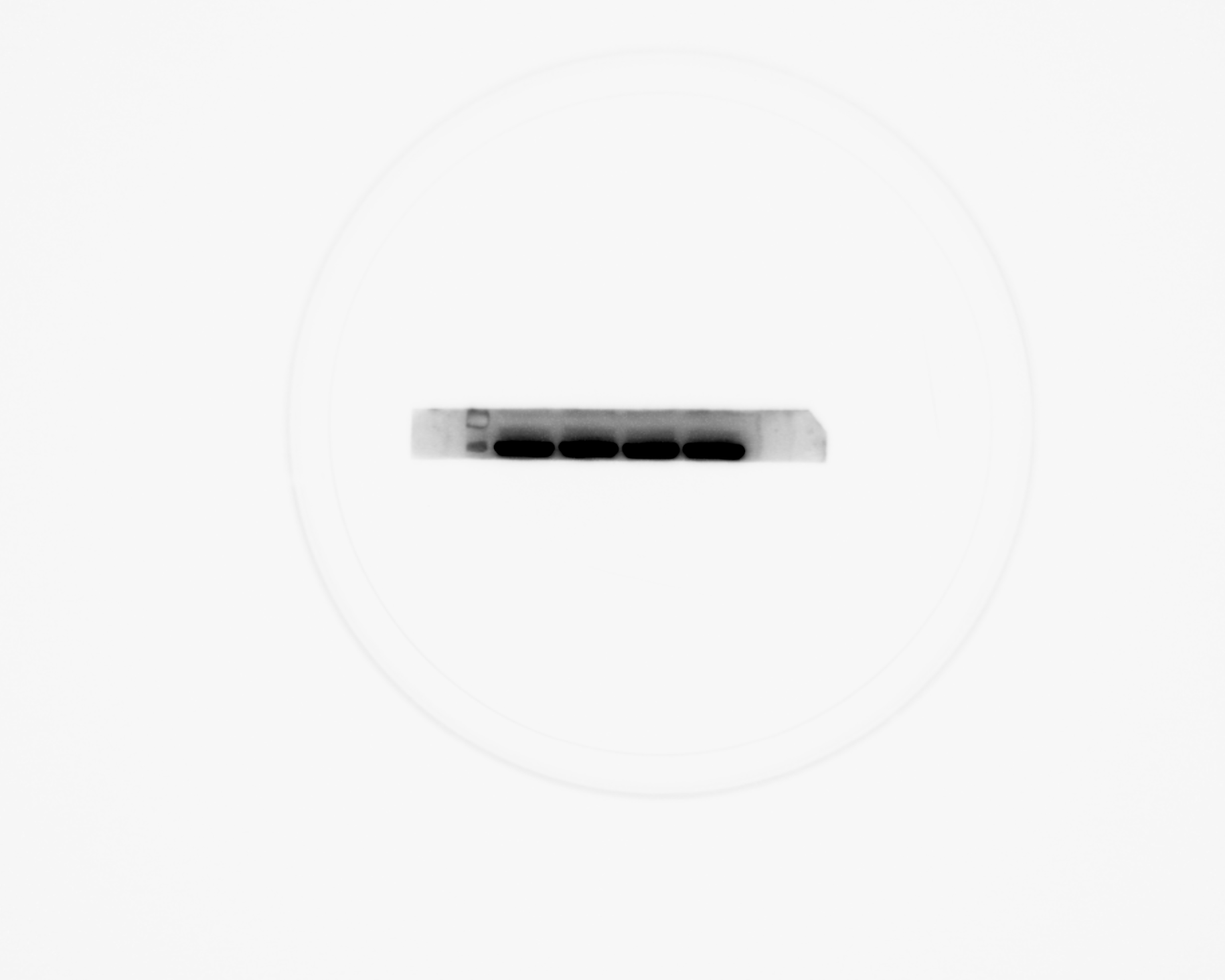

Supplement: Supplementary file 1 [file Presentation_1.zip › original bands for frontiers in pharmacology/alpha-tubulin original bands/alpha-tubulin-1.tif]

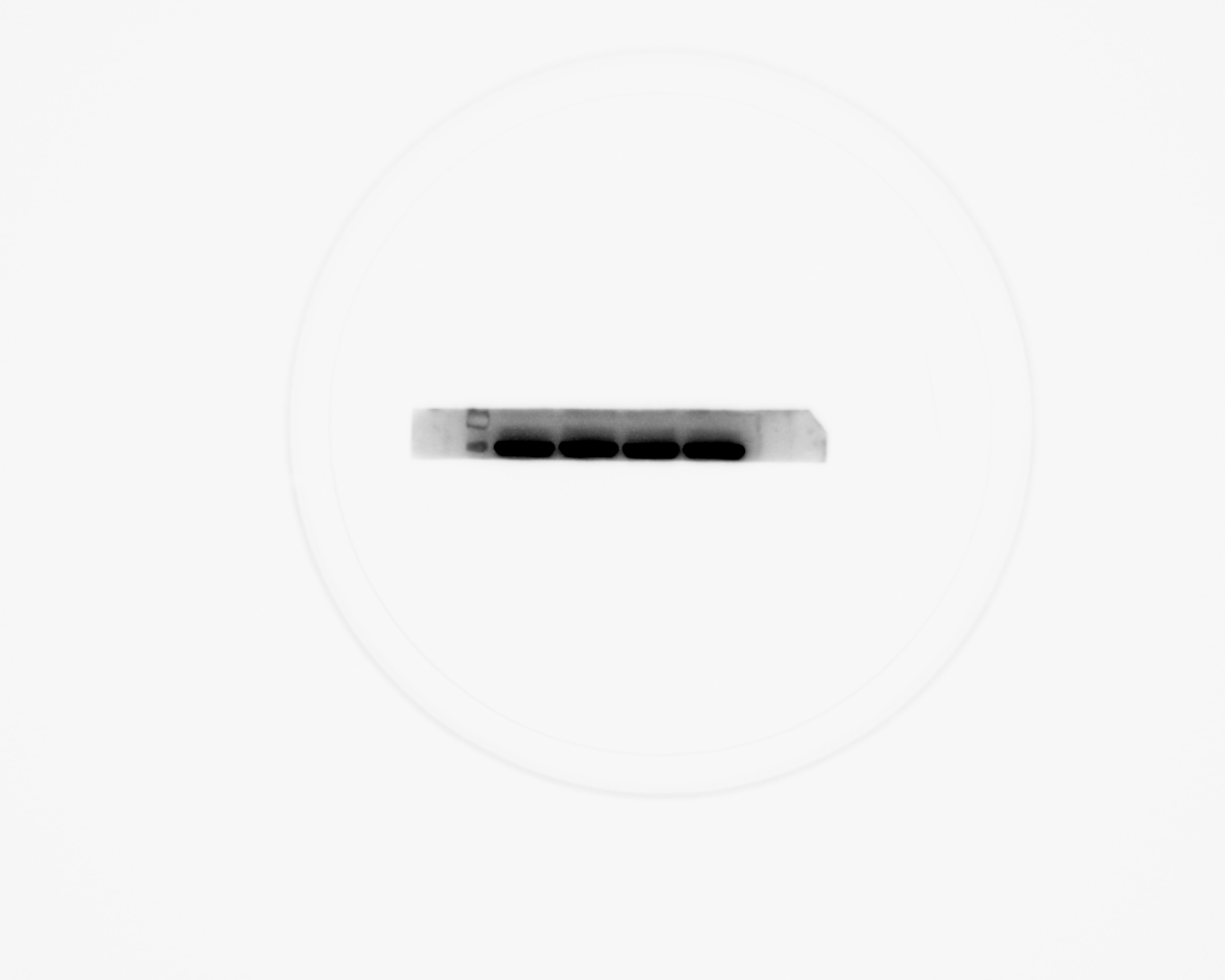

Supplement: Supplementary file 1 [file Presentation_1.zip › original bands for frontiers in pharmacology/alpha-tubulin original bands/alpha-tubulin-2.tif]

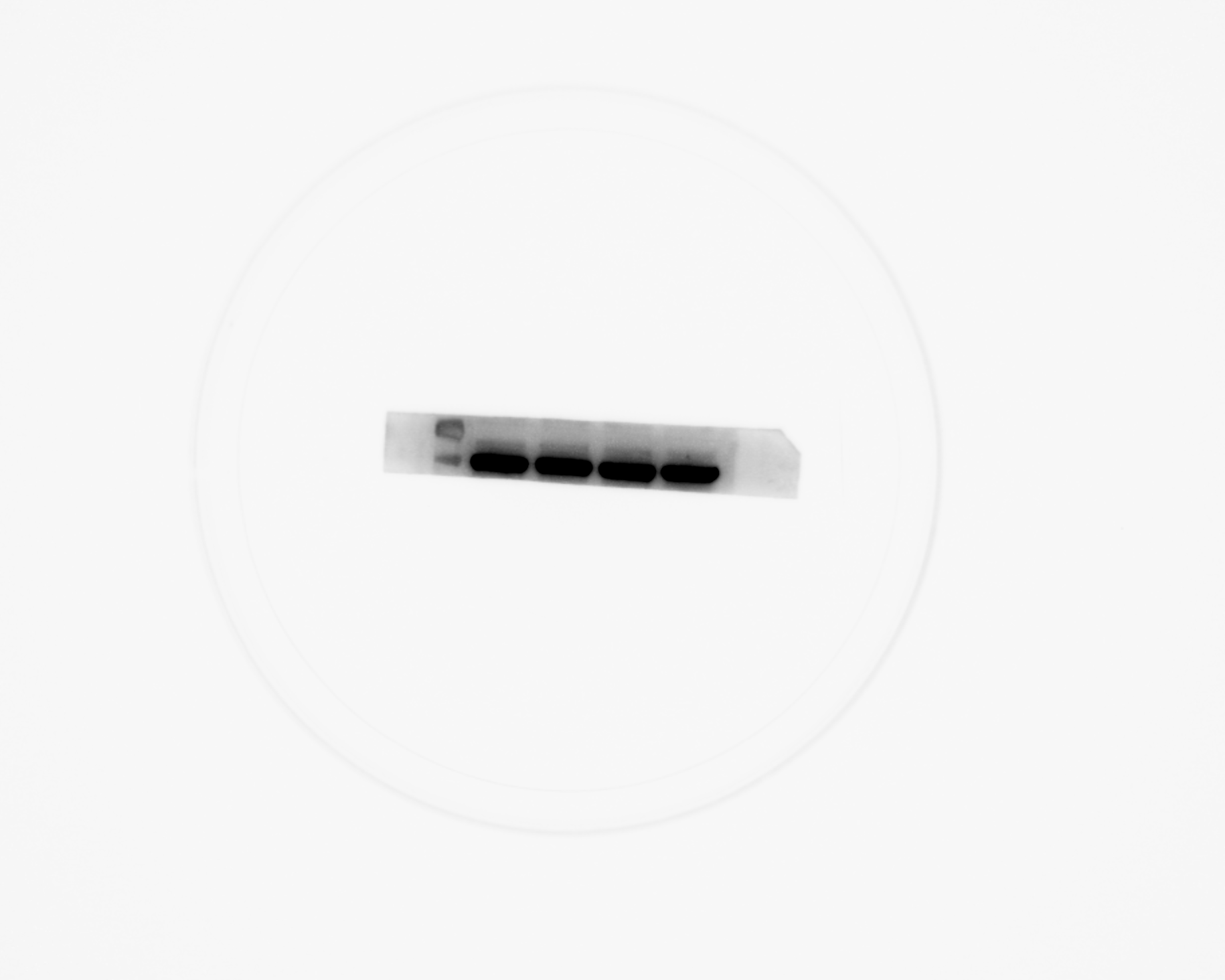

Supplement: Supplementary file 1 [file Presentation_1.zip › original bands for frontiers in pharmacology/alpha-tubulin original bands/alpha-tubulin-3 represent.tif]

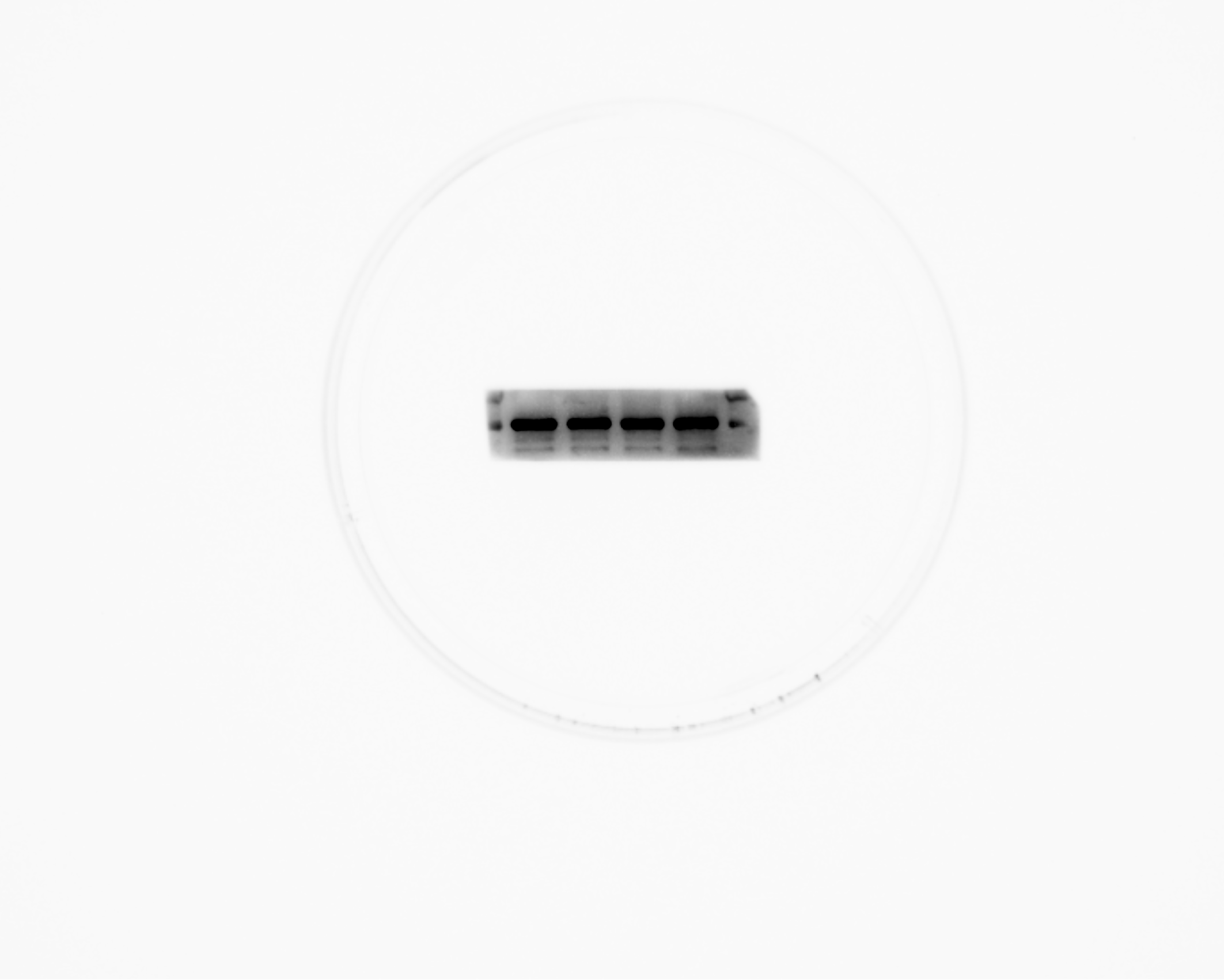

Supplement: Supplementary file 1 [file Presentation_1.zip › original bands for frontiers in pharmacology/alpha-tubulin original bands/alpha-tubulin-4.tif]

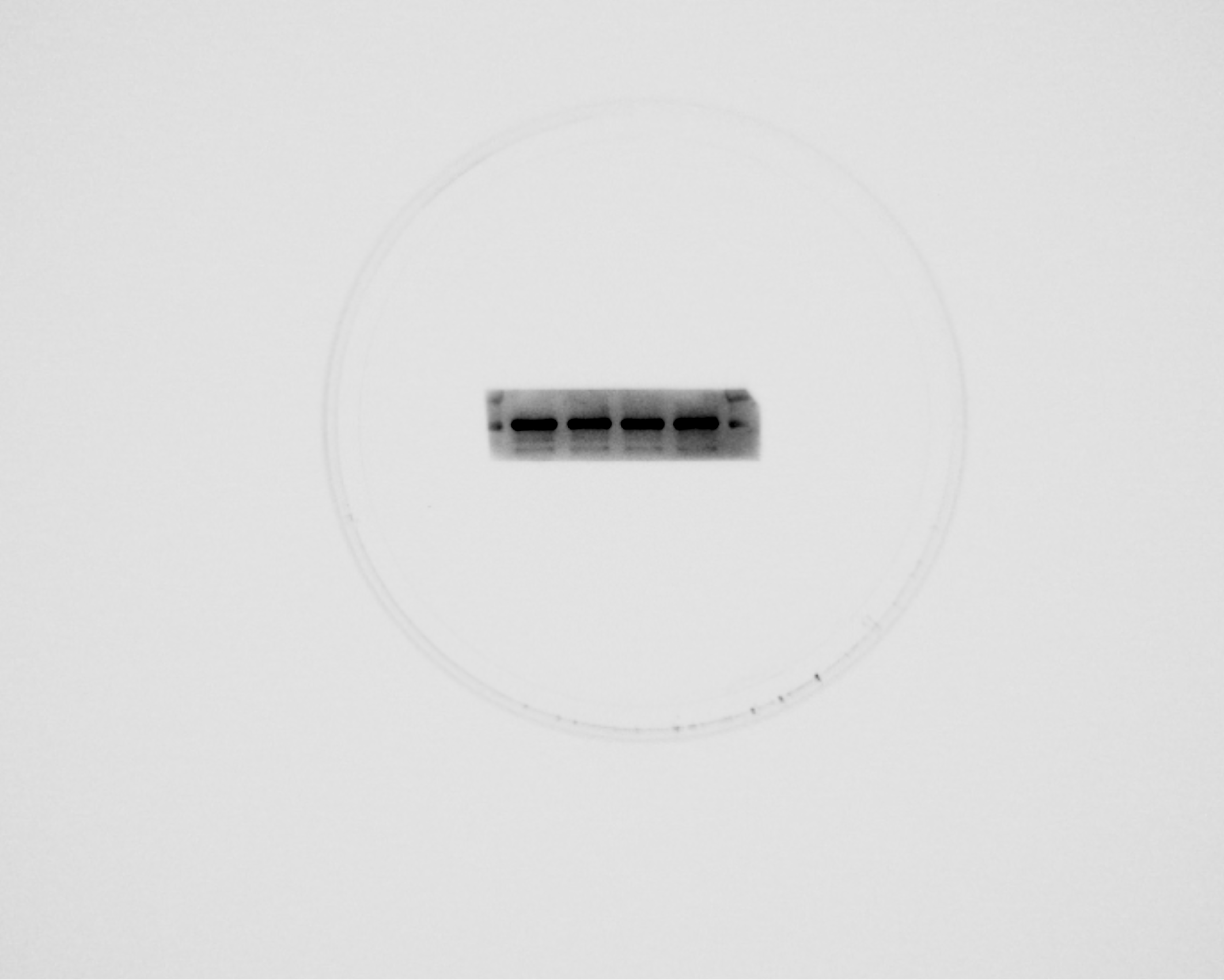

Supplement: Supplementary file 1 [file Presentation_1.zip › original bands for frontiers in pharmacology/alpha-tubulin original bands/alpha-tubulin-5.tif]

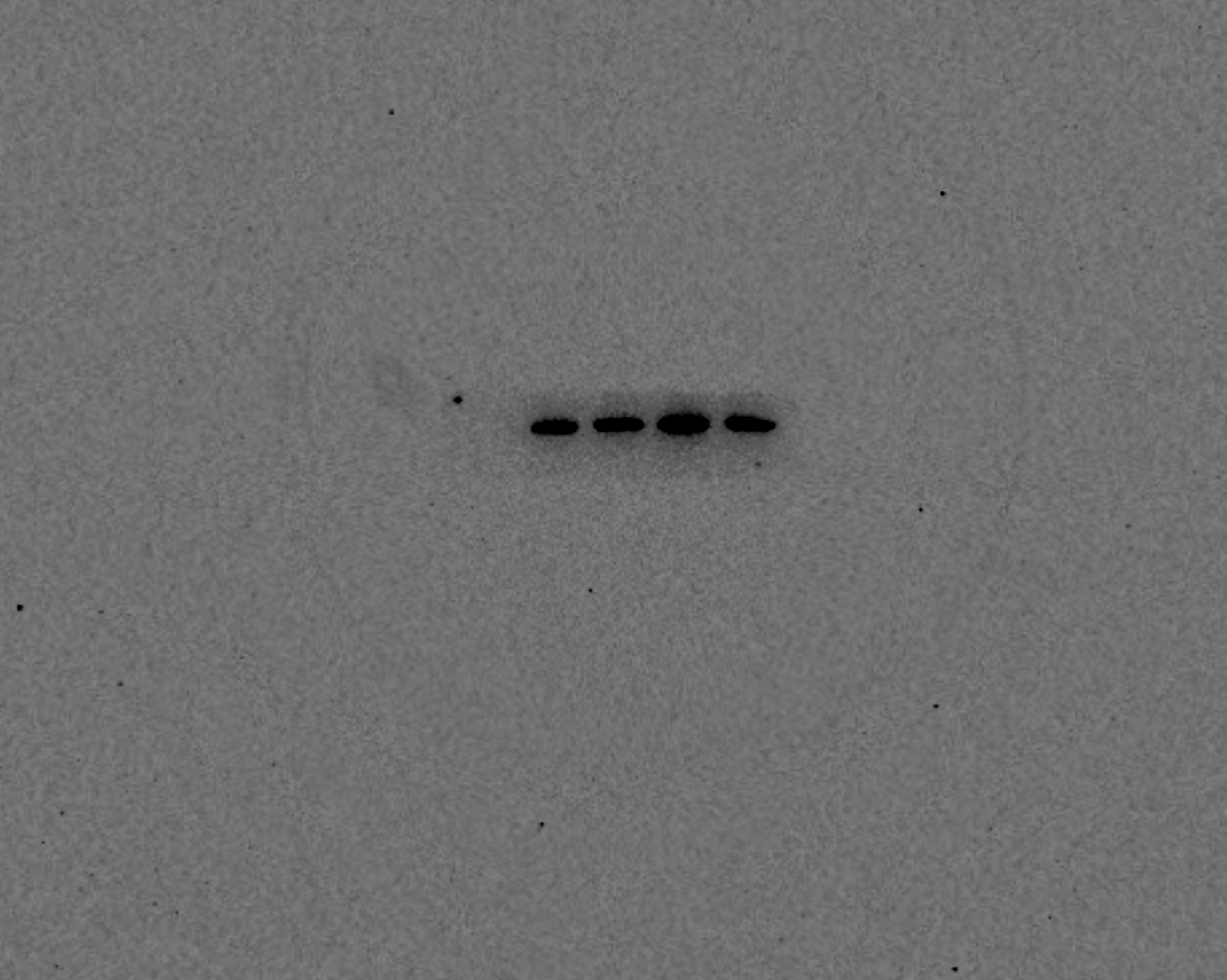

Supplement: Supplementary file 1 [file Presentation_1.zip › original bands for frontiers in pharmacology/Bax original bands/Bax-1.tif]

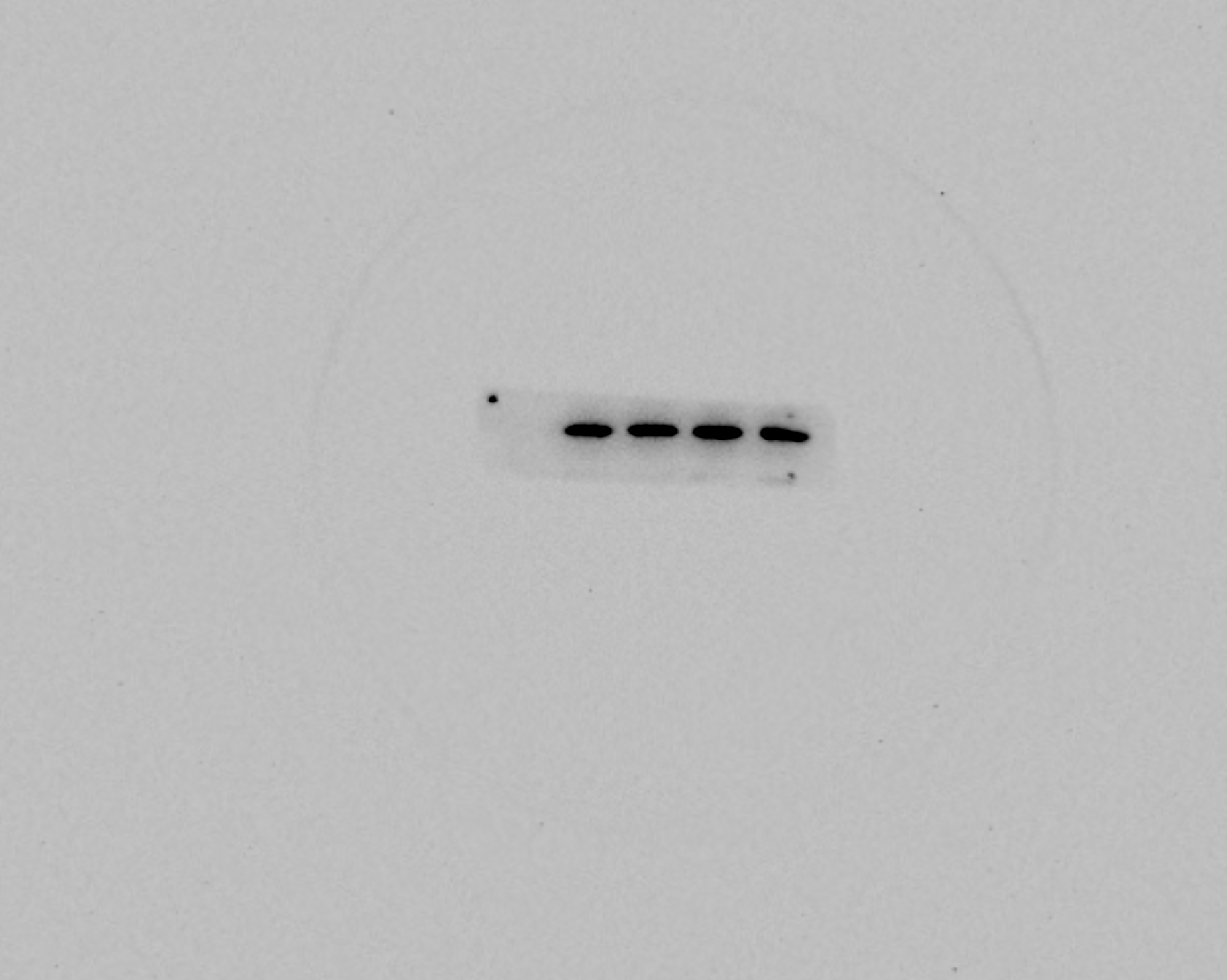

Supplement: Supplementary file 1 [file Presentation_1.zip › original bands for frontiers in pharmacology/Bax original bands/Bax-2.tif]

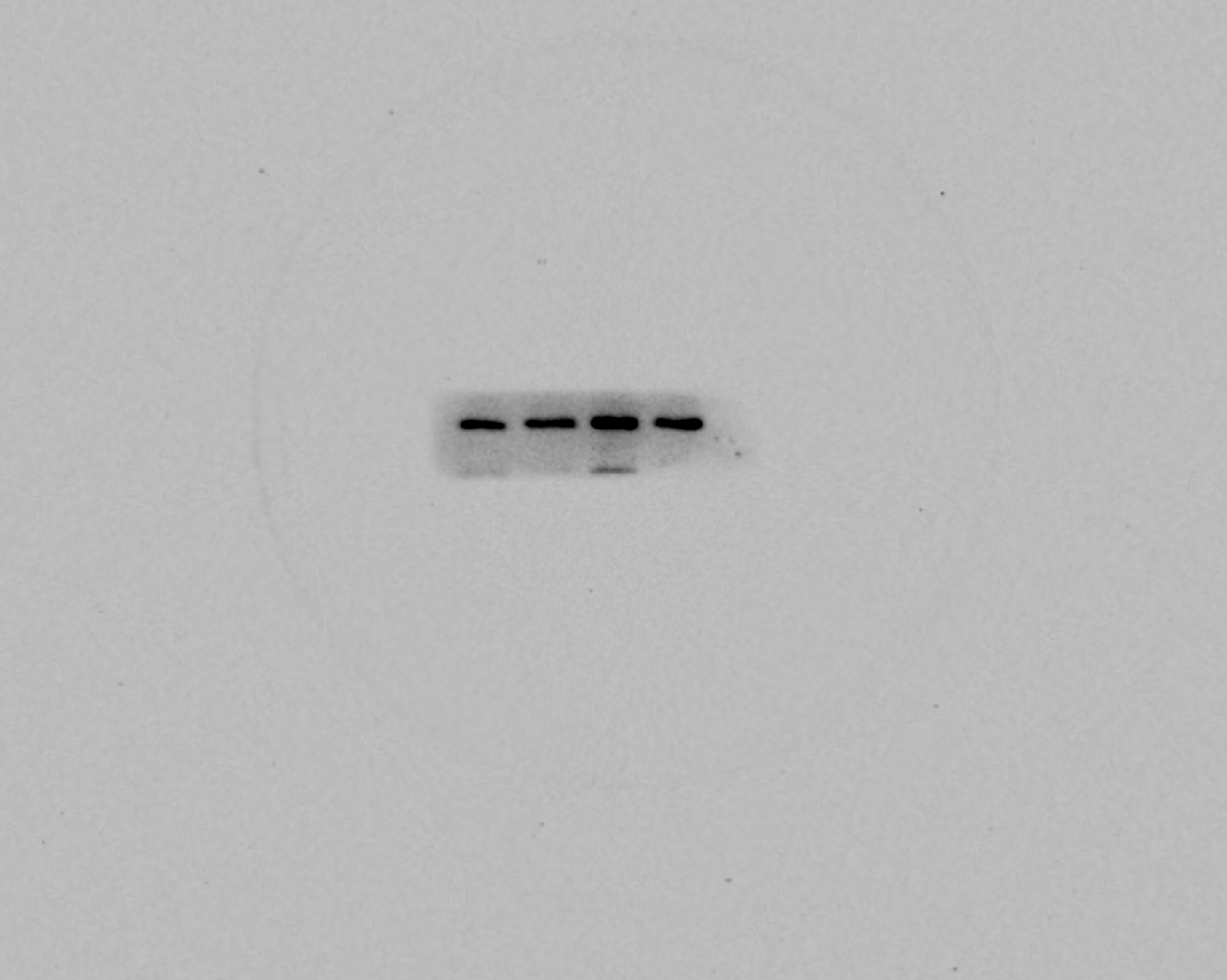

Supplement: Supplementary file 1 [file Presentation_1.zip › original bands for frontiers in pharmacology/Bax original bands/Bax-3.tif]

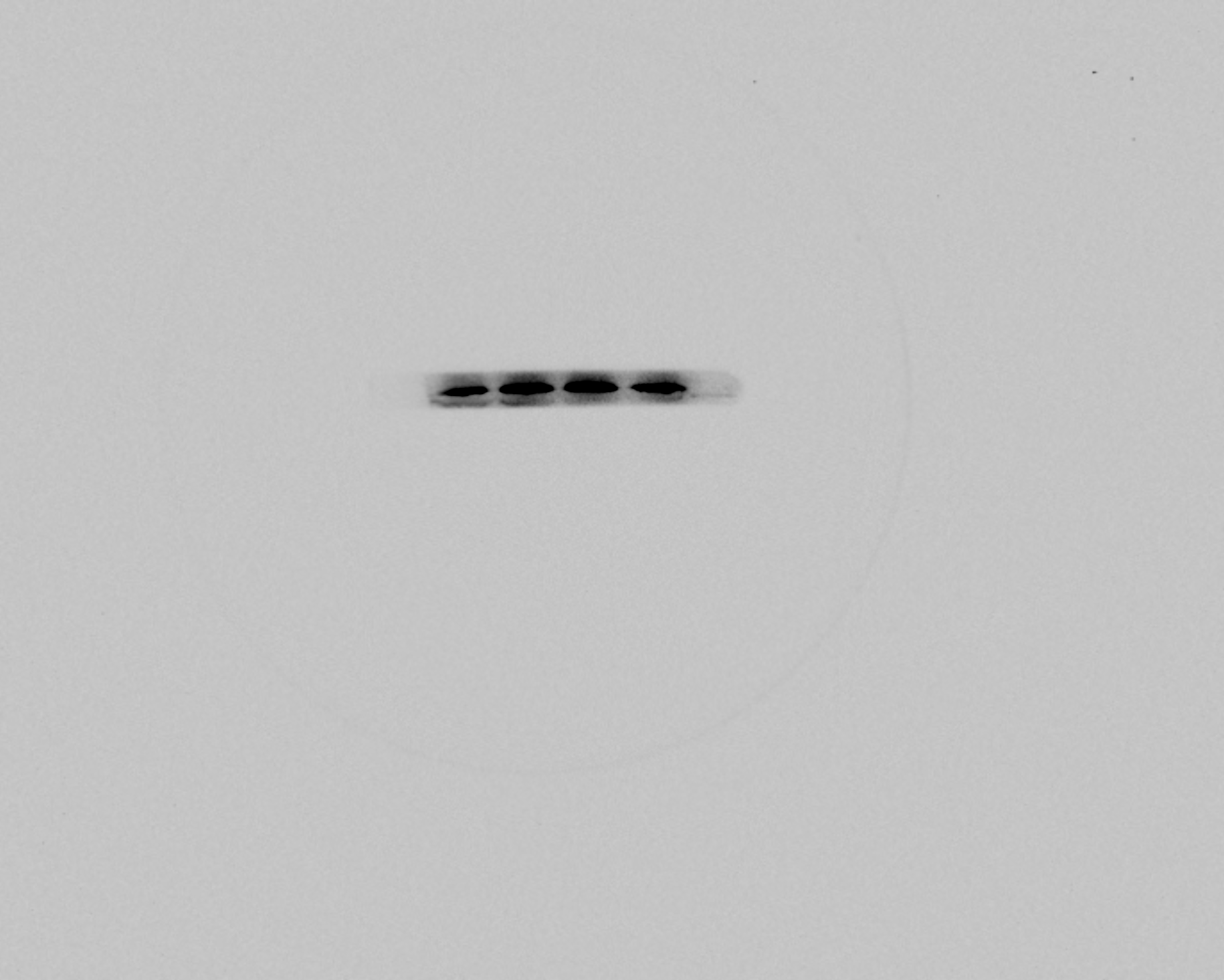

Supplement: Supplementary file 1 [file Presentation_1.zip › original bands for frontiers in pharmacology/Bax original bands/Bax-4.tif]

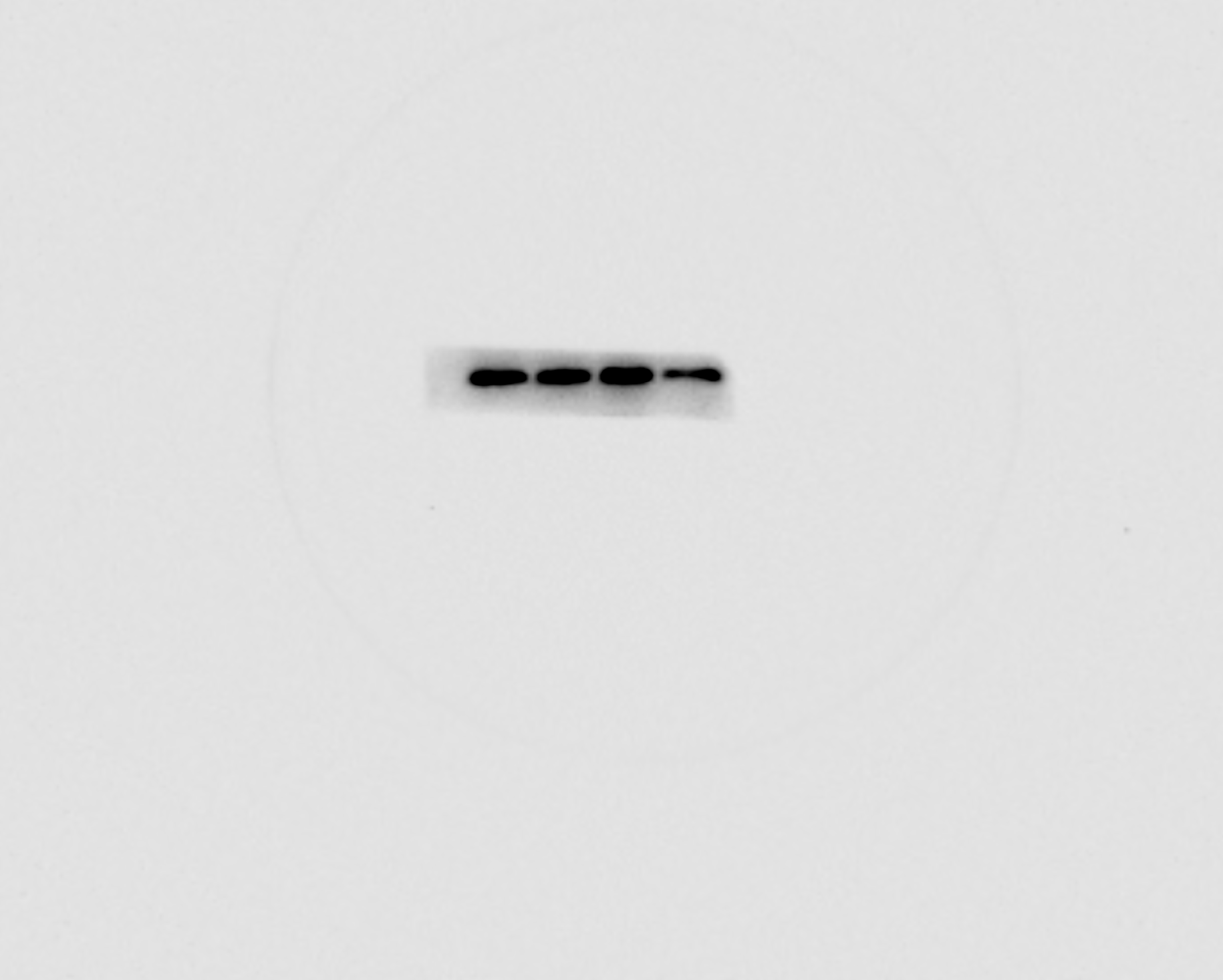

Supplement: Supplementary file 1 [file Presentation_1.zip › original bands for frontiers in pharmacology/Bax original bands/Bax-5.tif]

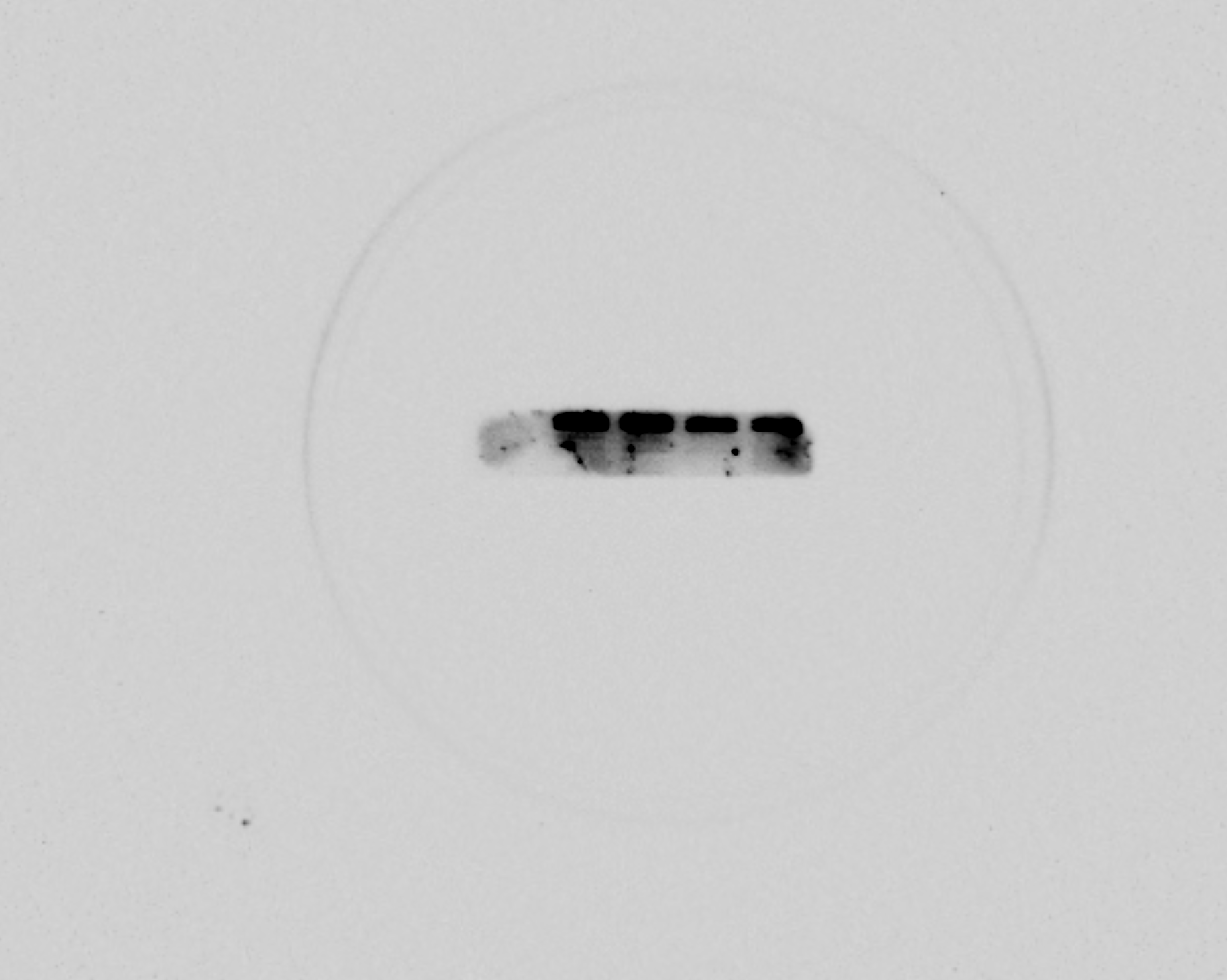

Supplement: Supplementary file 1 [file Presentation_1.zip › original bands for frontiers in pharmacology/Bcl-2 original bands/Bcl-2-1 represent.tif]

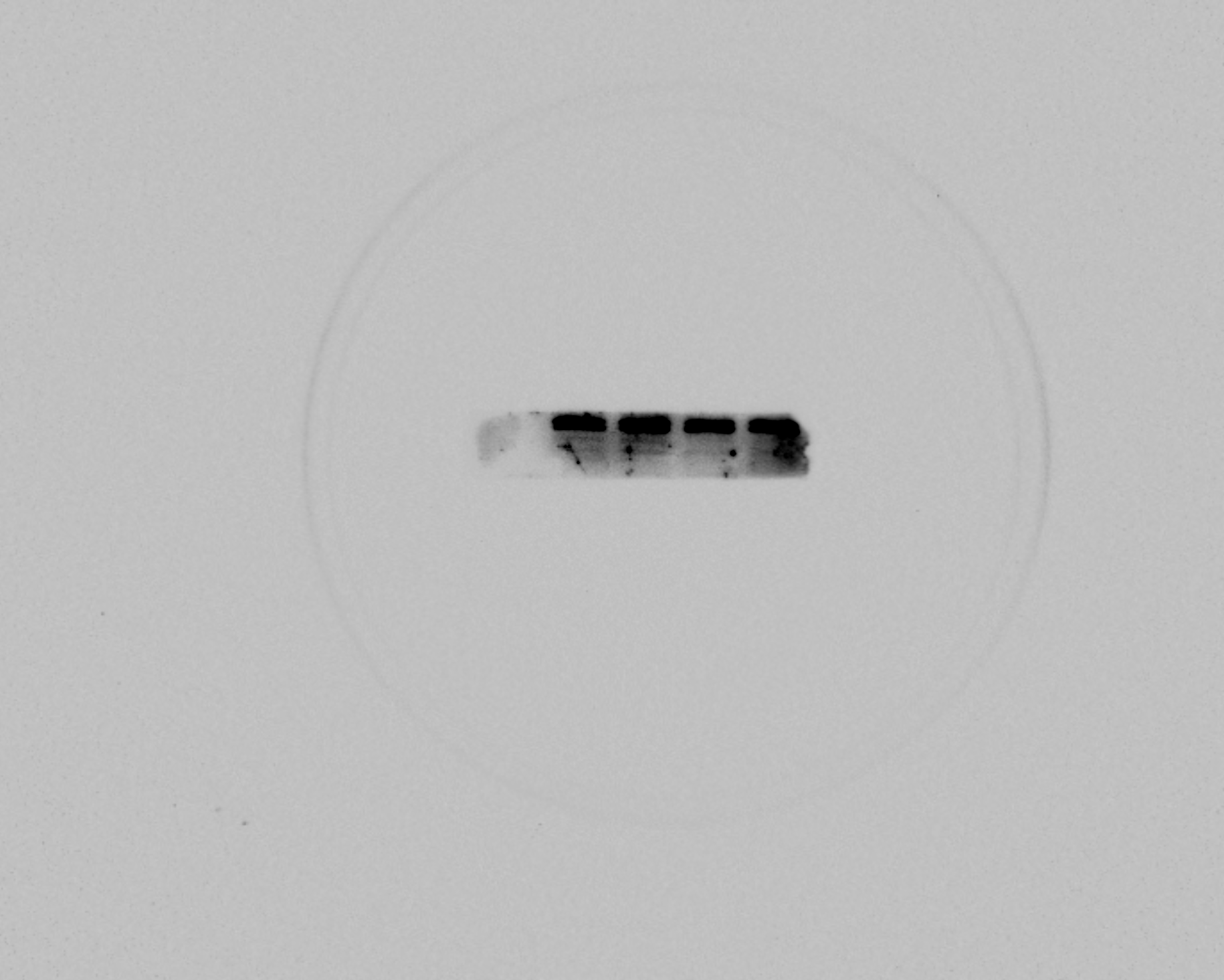

Supplement: Supplementary file 1 [file Presentation_1.zip › original bands for frontiers in pharmacology/Bcl-2 original bands/Bcl-2-2.tif]

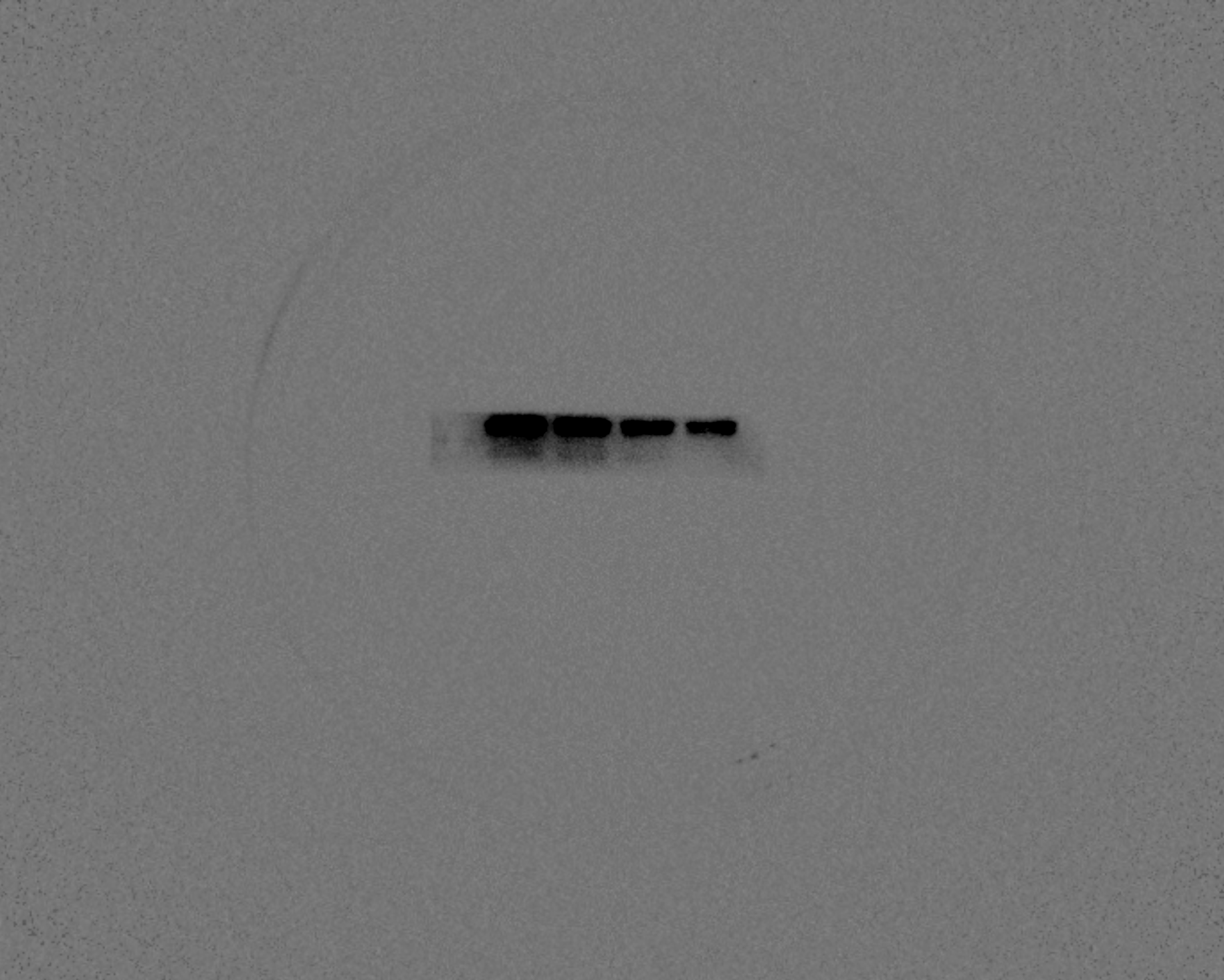

Supplement: Supplementary file 1 [file Presentation_1.zip › original bands for frontiers in pharmacology/Bcl-2 original bands/Bcl-2-3.tif]

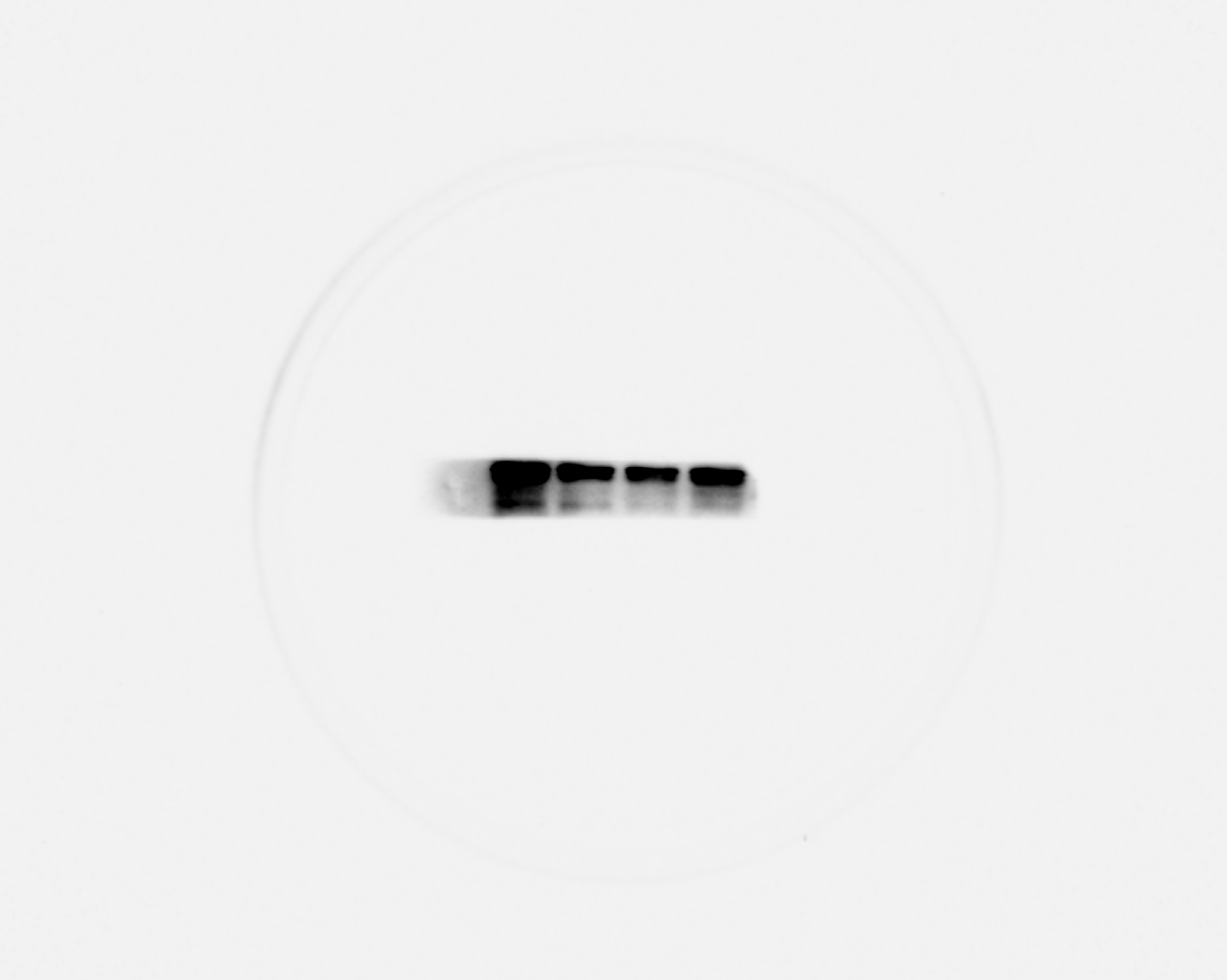

Supplement: Supplementary file 1 [file Presentation_1.zip › original bands for frontiers in pharmacology/Bcl-2 original bands/Bcl-2-4.tif]

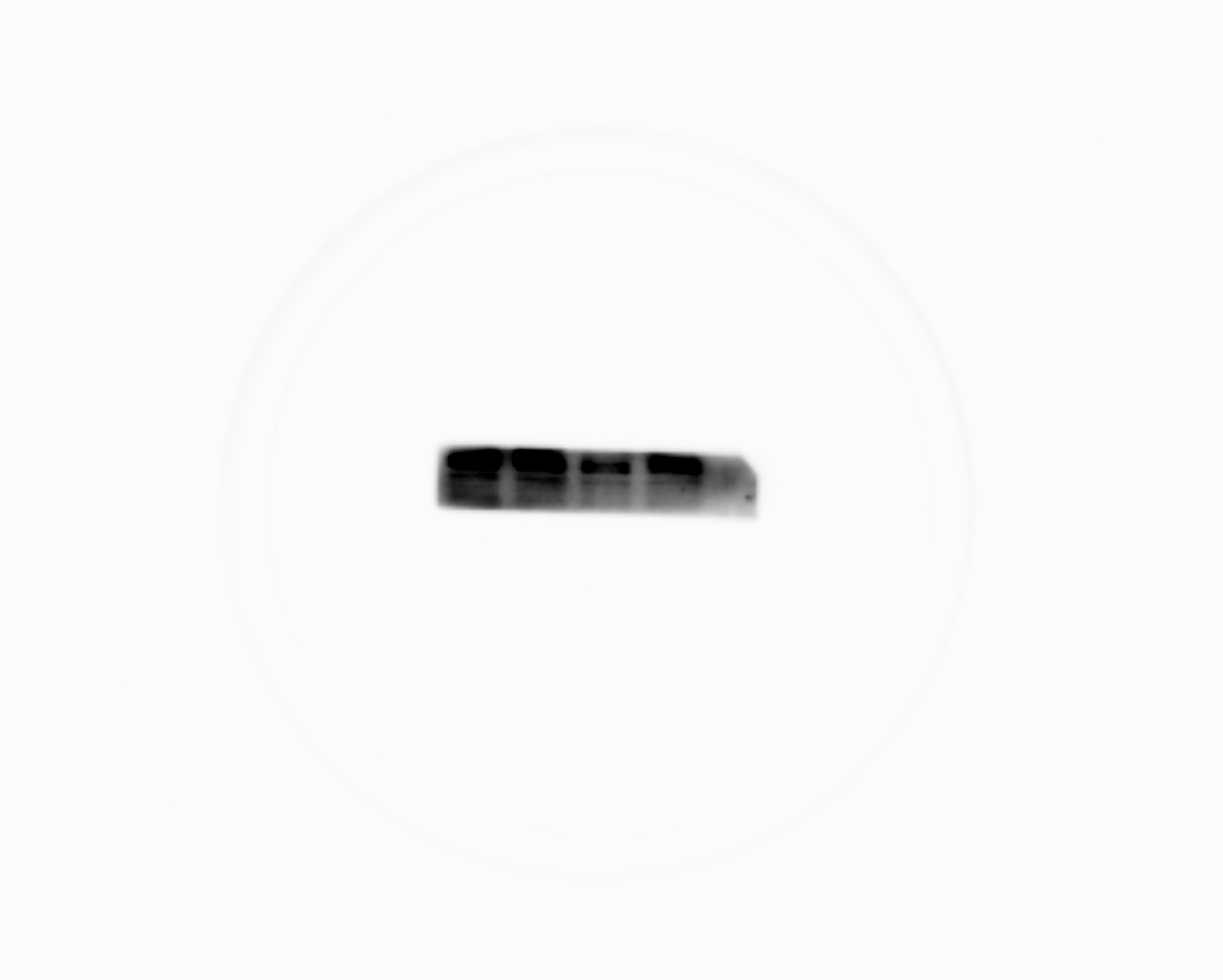

Supplement: Supplementary file 1 [file Presentation_1.zip › original bands for frontiers in pharmacology/Bcl-2 original bands/Bcl-2-5.tif]

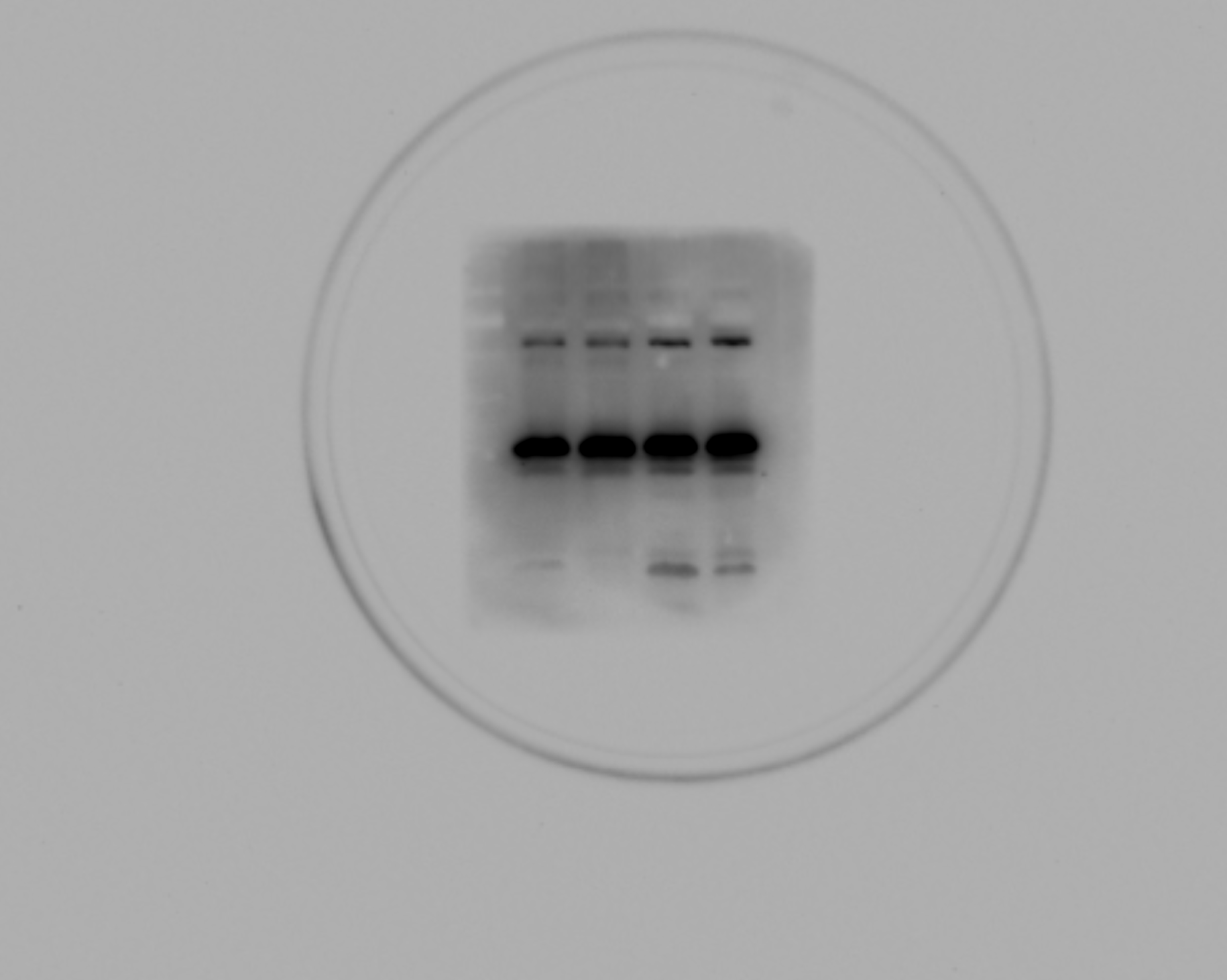

Supplement: Supplementary file 1 [file Presentation_1.zip › original bands for frontiers in pharmacology/caspase-3 original bands/caspase-3-1.tif]

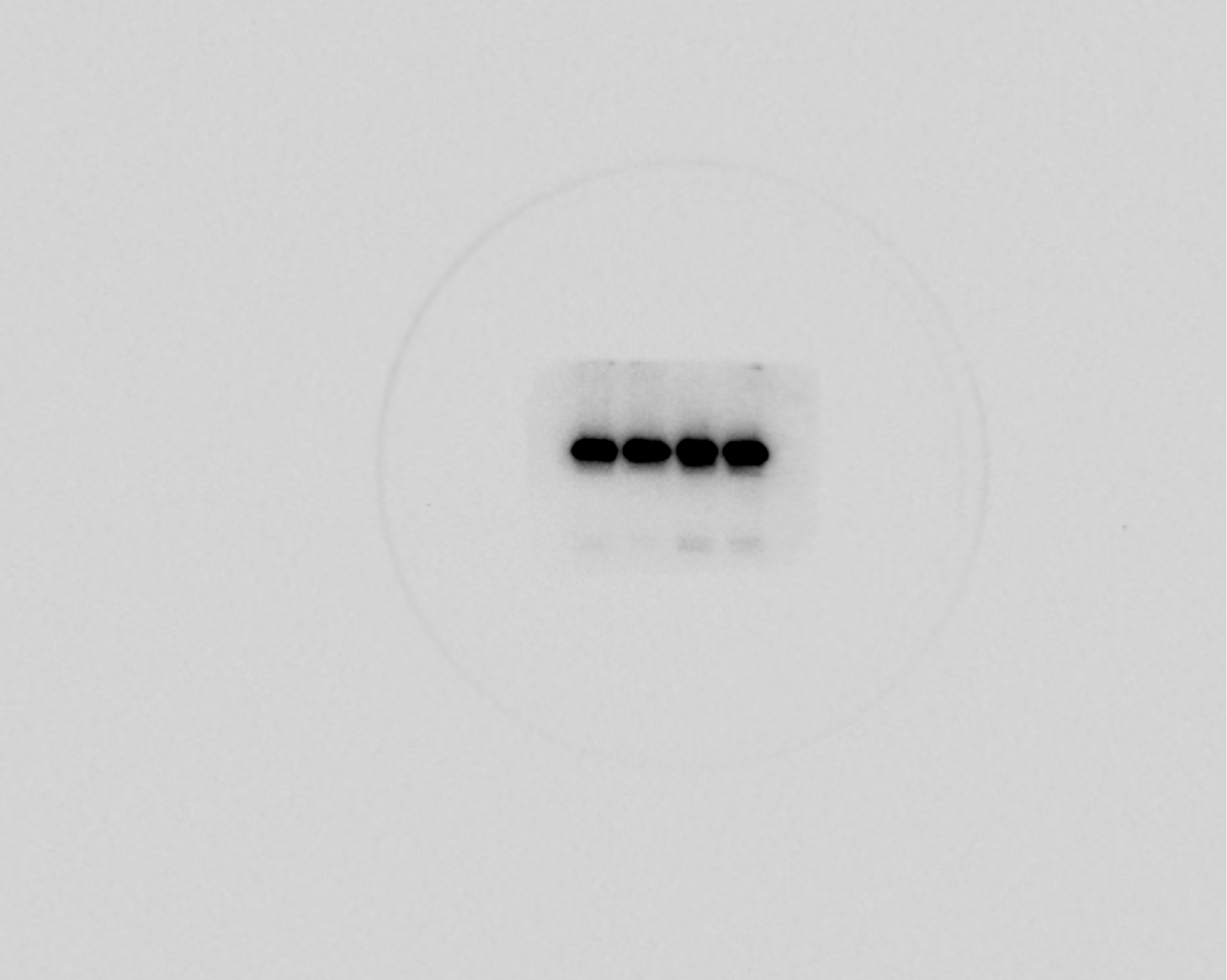

Supplement: Supplementary file 1 [file Presentation_1.zip › original bands for frontiers in pharmacology/caspase-3 original bands/caspase-3-2.tif]

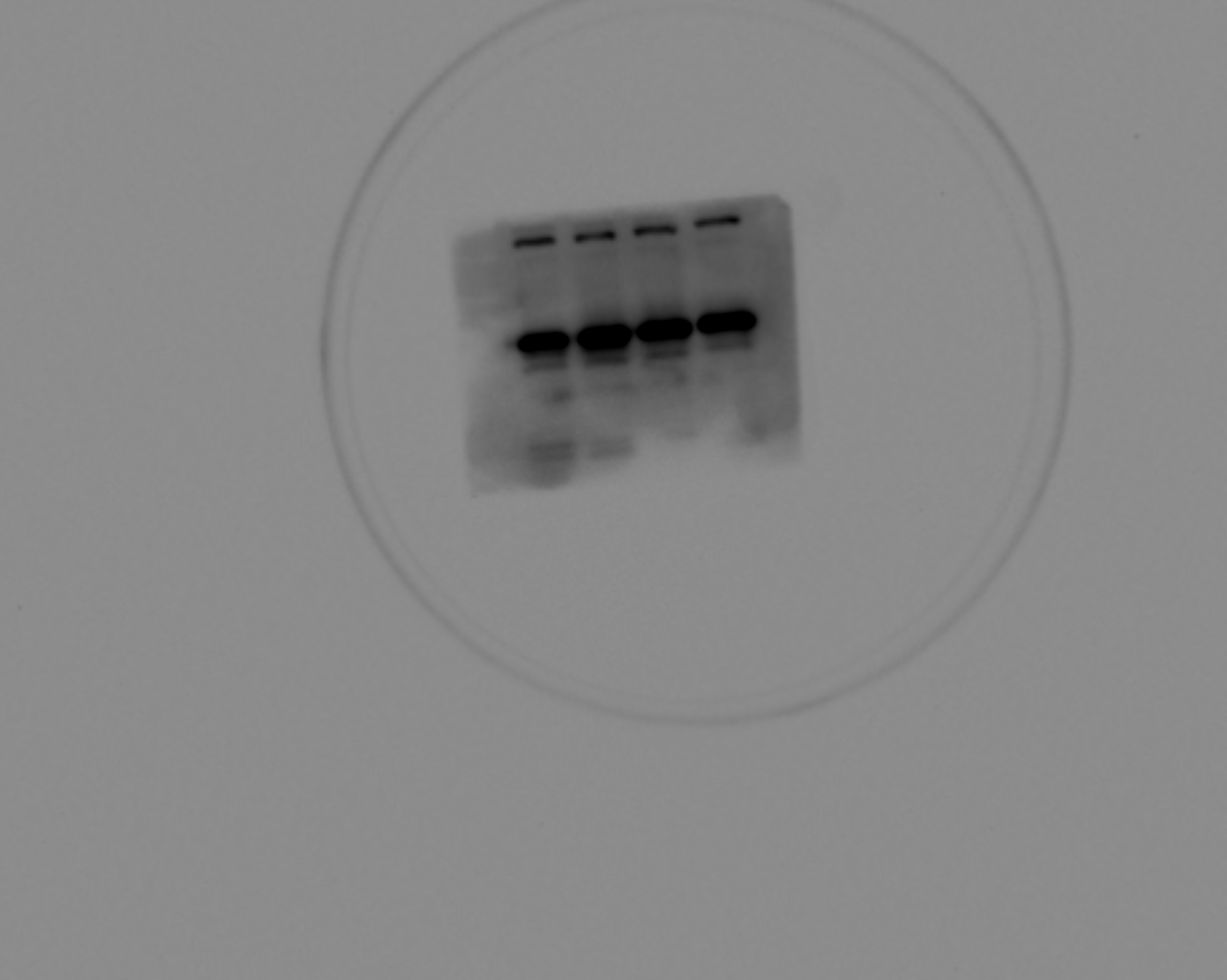

Supplement: Supplementary file 1 [file Presentation_1.zip › original bands for frontiers in pharmacology/caspase-3 original bands/caspase-3-3.tif]

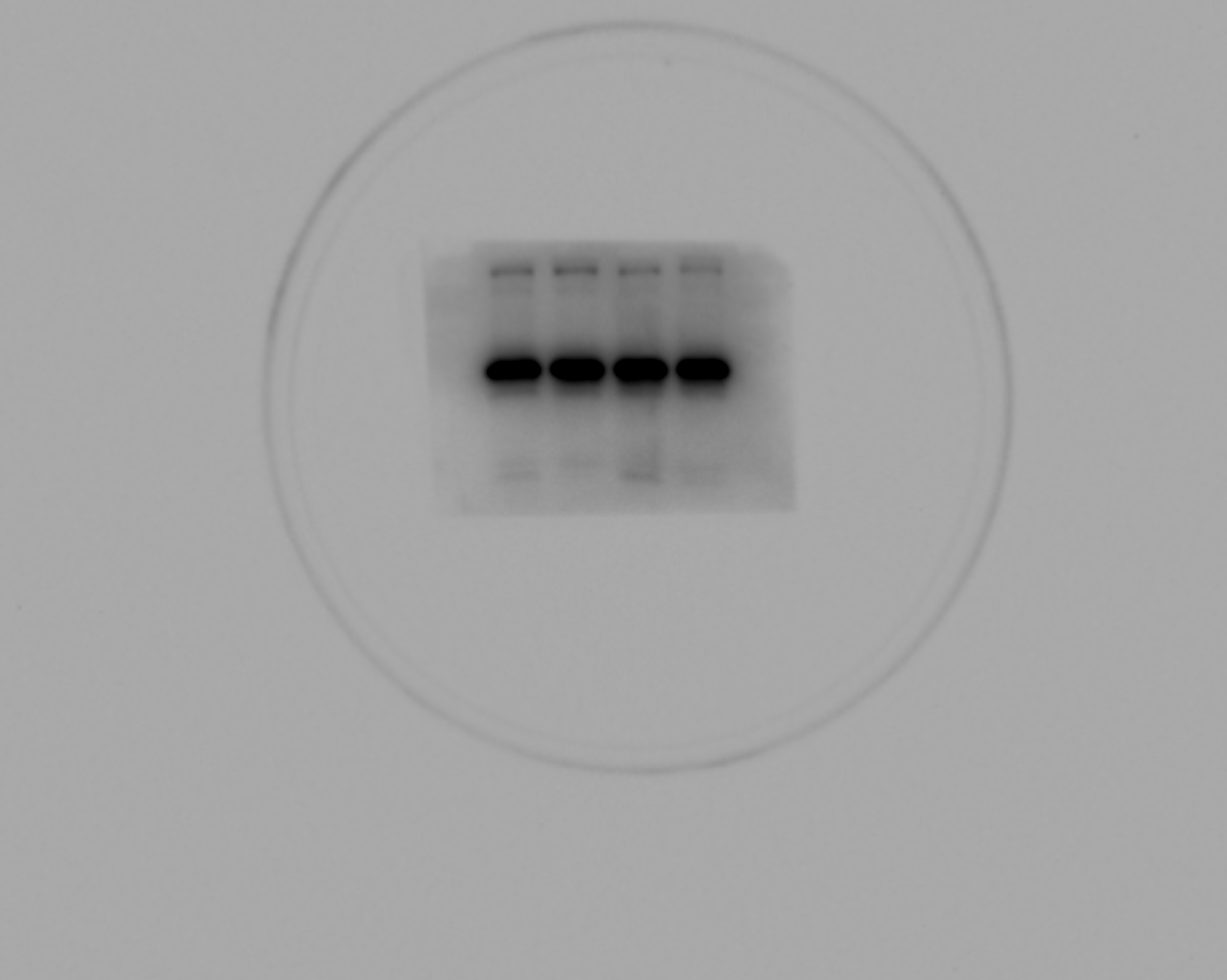

Supplement: Supplementary file 1 [file Presentation_1.zip › original bands for frontiers in pharmacology/caspase-3 original bands/caspase-3-4 represent.tif]

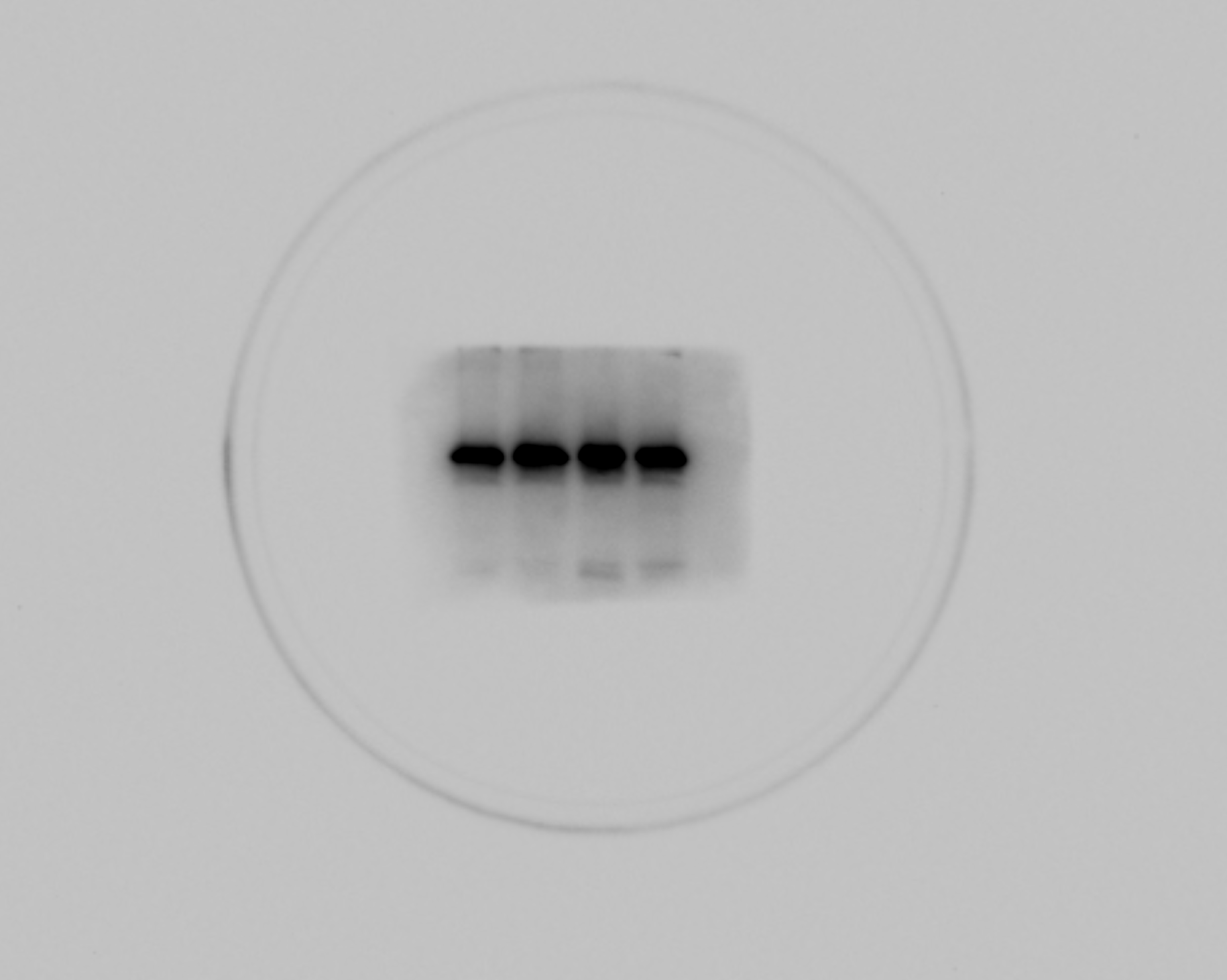

Supplement: Supplementary file 1 [file Presentation_1.zip › original bands for frontiers in pharmacology/caspase-3 original bands/caspase-3-5.tif]

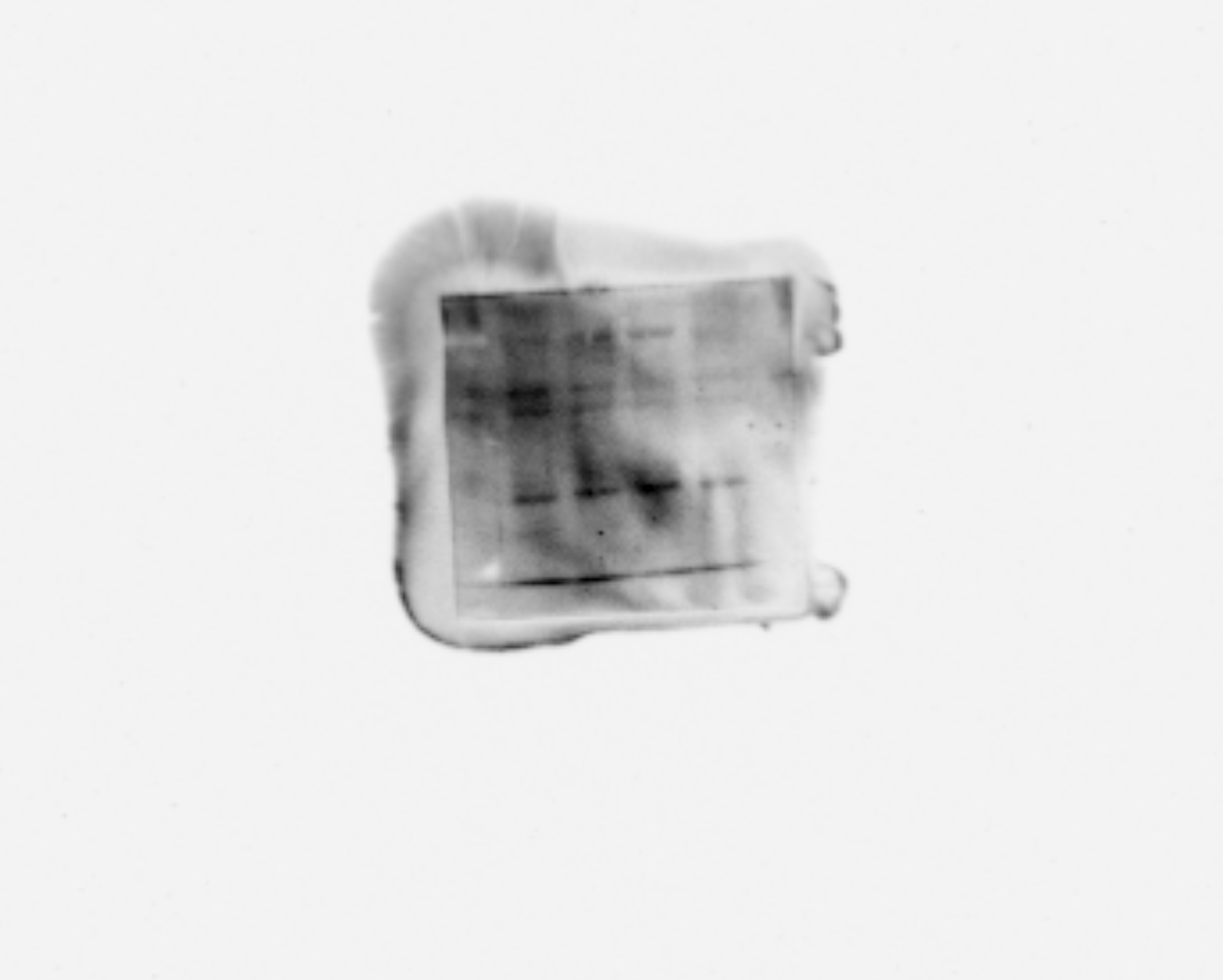

Supplement: Supplementary file 1 [file Presentation_1.zip › original bands for frontiers in pharmacology/cleaved-caspase-3 original bands/cleaved-caspase-3-1.tif]

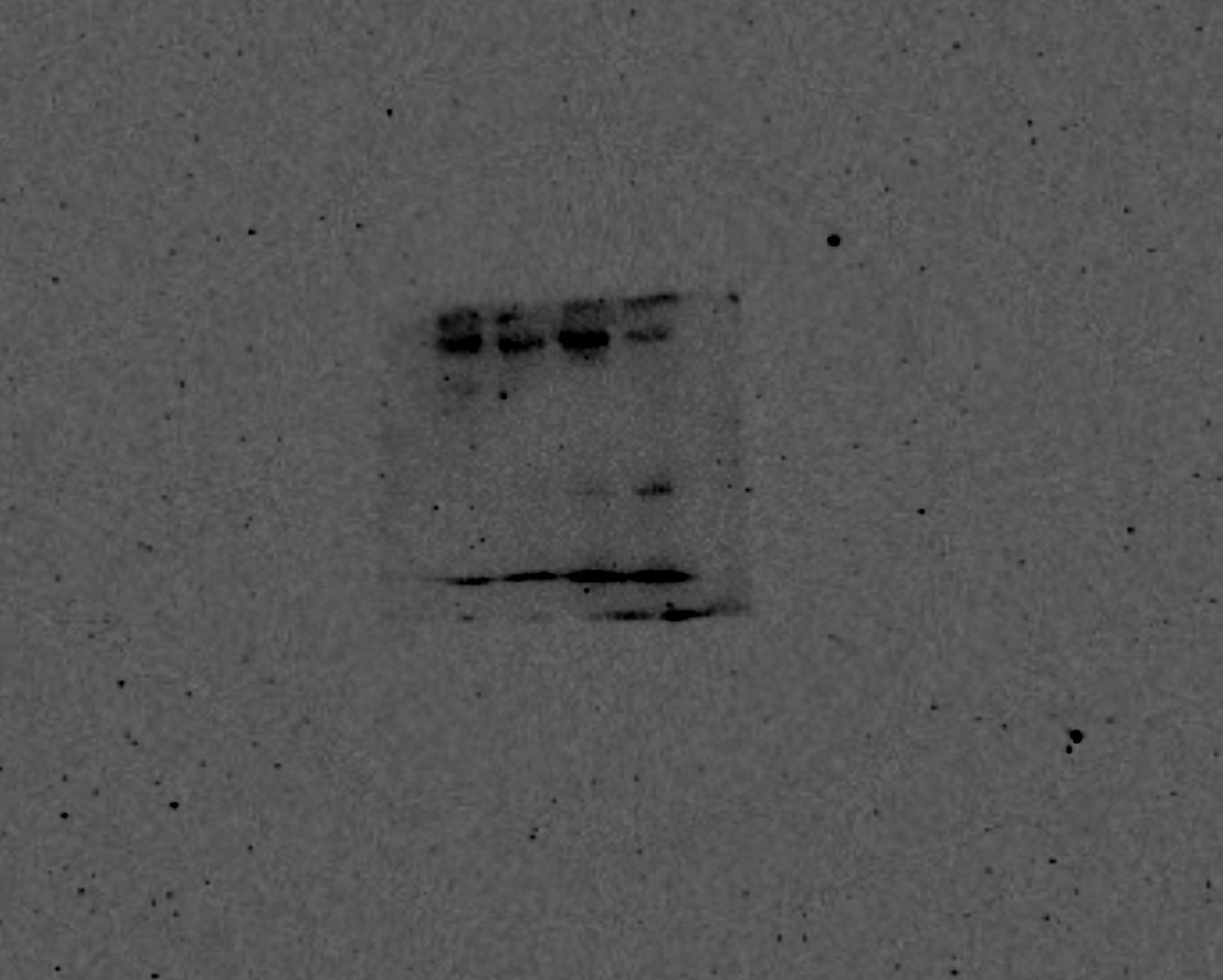

Supplement: Supplementary file 1 [file Presentation_1.zip › original bands for frontiers in pharmacology/cleaved-caspase-3 original bands/cleaved-caspase-3-2.tif]

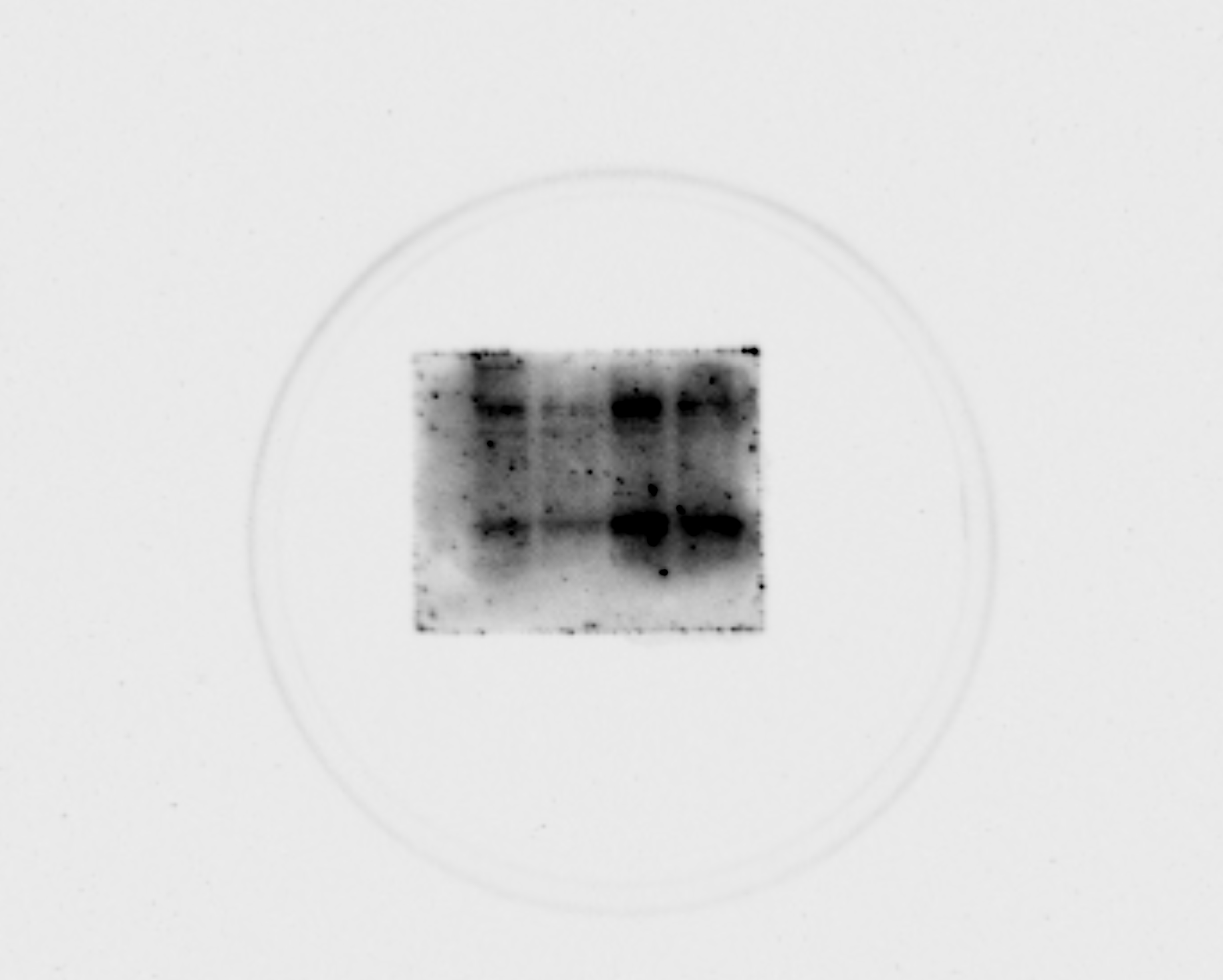

Supplement: Supplementary file 1 [file Presentation_1.zip › original bands for frontiers in pharmacology/cleaved-caspase-3 original bands/cleaved-caspase-3-3.tif]

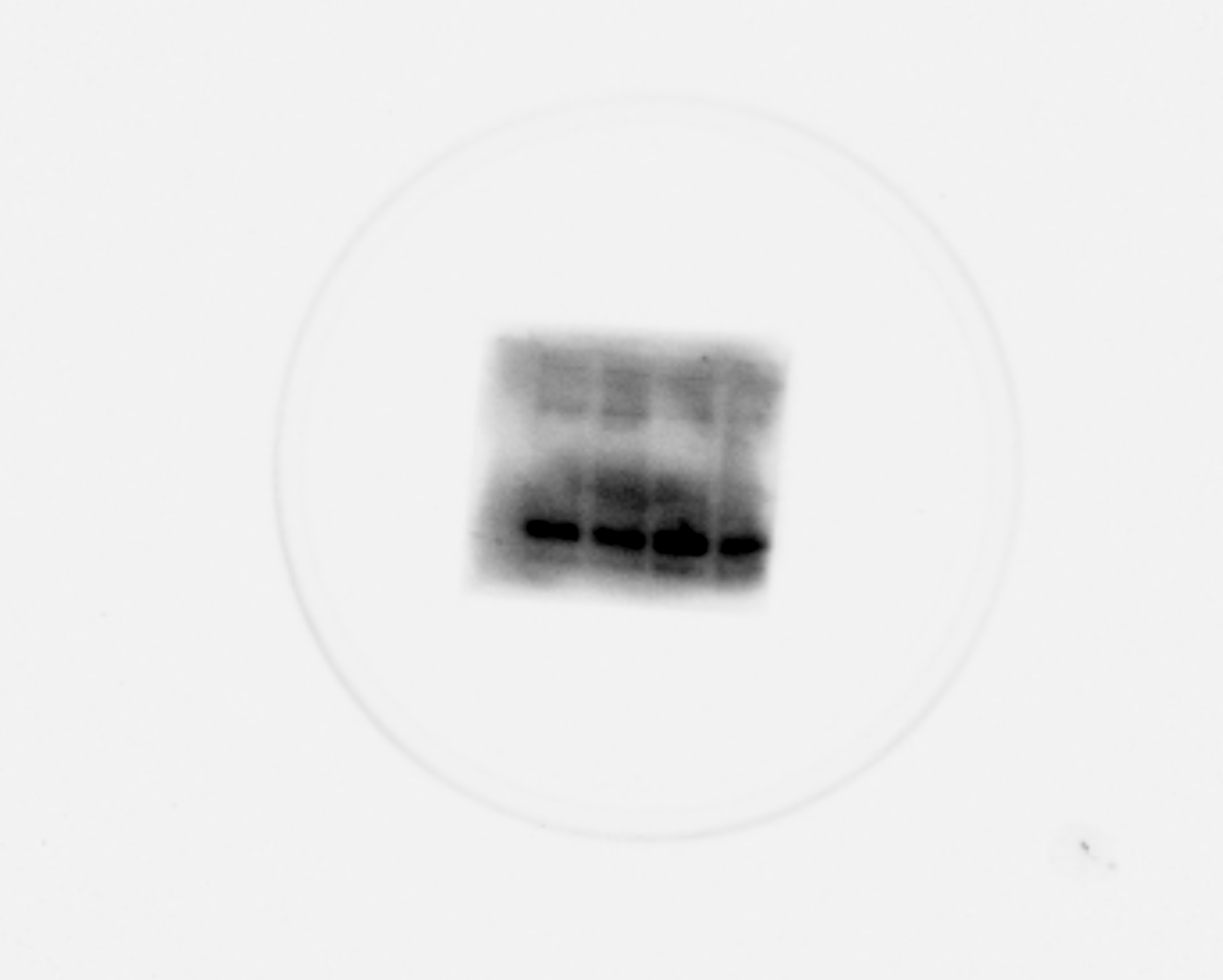

Supplement: Supplementary file 1 [file Presentation_1.zip › original bands for frontiers in pharmacology/cleaved-caspase-3 original bands/cleaved-caspase-3-4.tif]

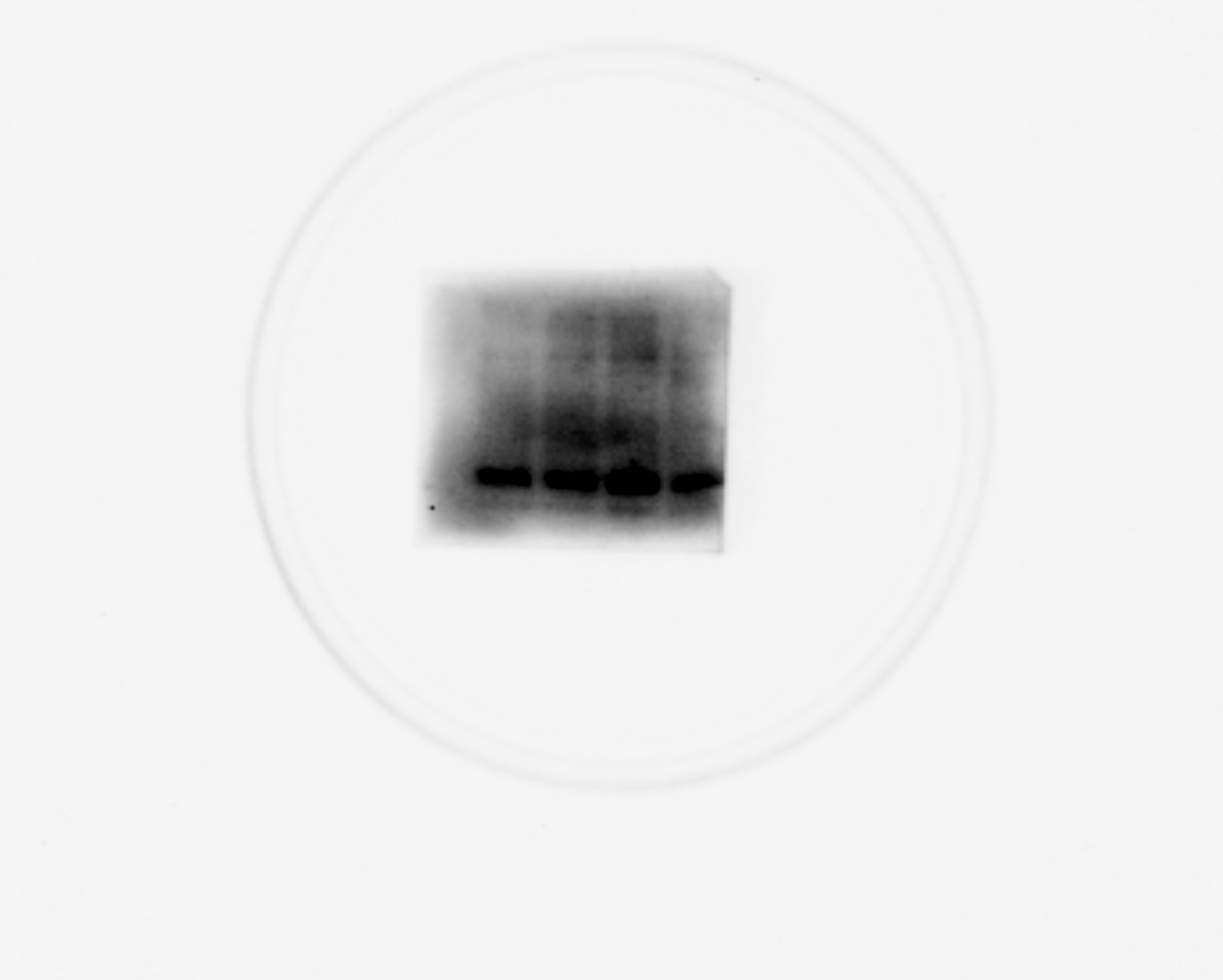

Supplement: Supplementary file 1 [file Presentation_1.zip › original bands for frontiers in pharmacology/cleaved-caspase-3 original bands/cleaved-caspase-3-5 represent.tif]

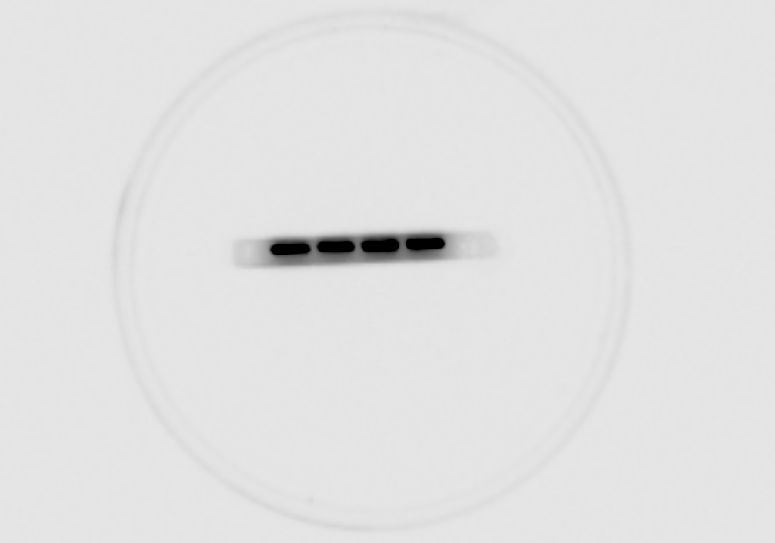

Supplement: Supplementary file 1 [file Presentation_1.zip › original bands for frontiers in pharmacology/GAPDH original bands/GAPDH-1 represent.tif]

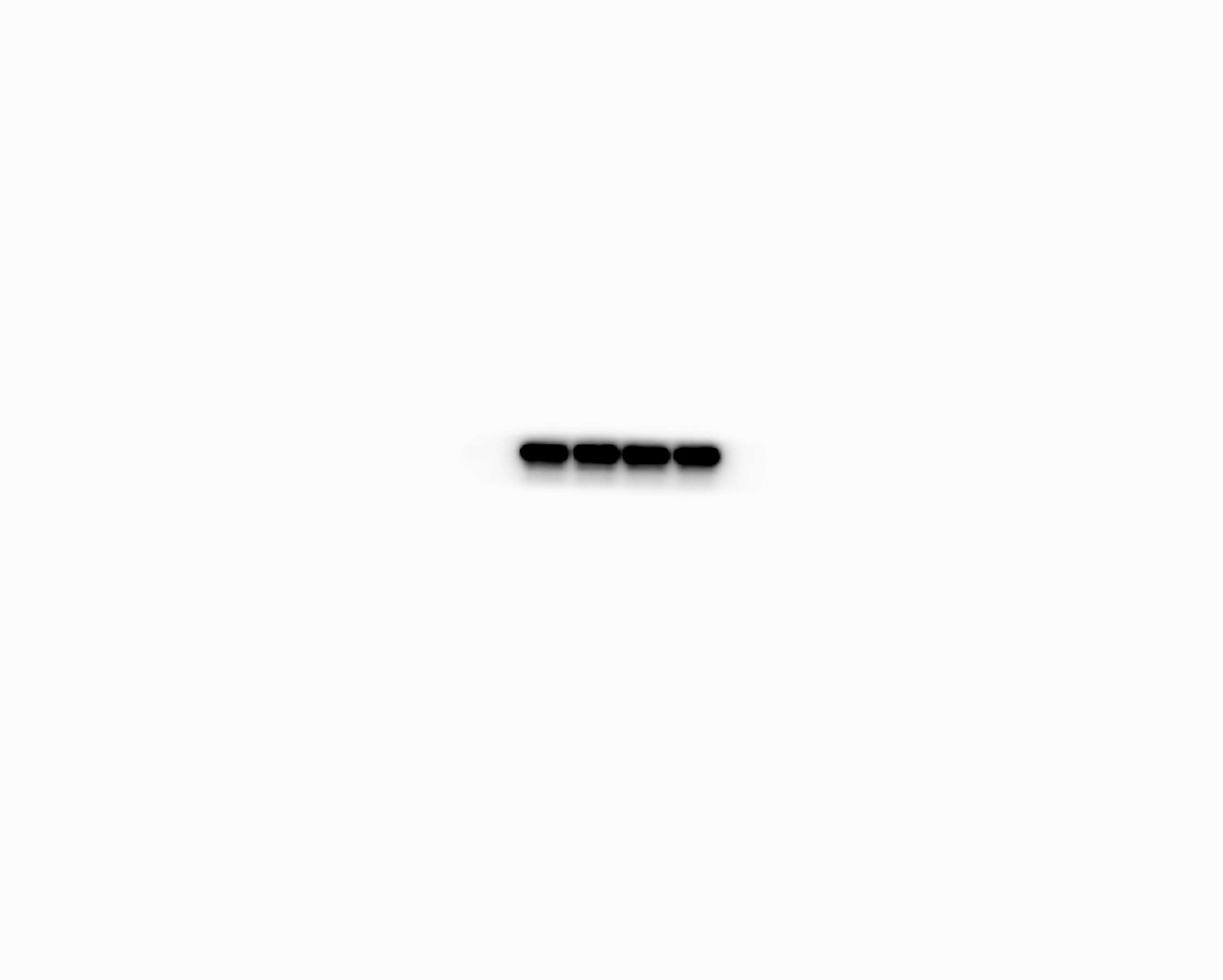

Supplement: Supplementary file 1 [file Presentation_1.zip › original bands for frontiers in pharmacology/GAPDH original bands/GAPDH-2.jpg]

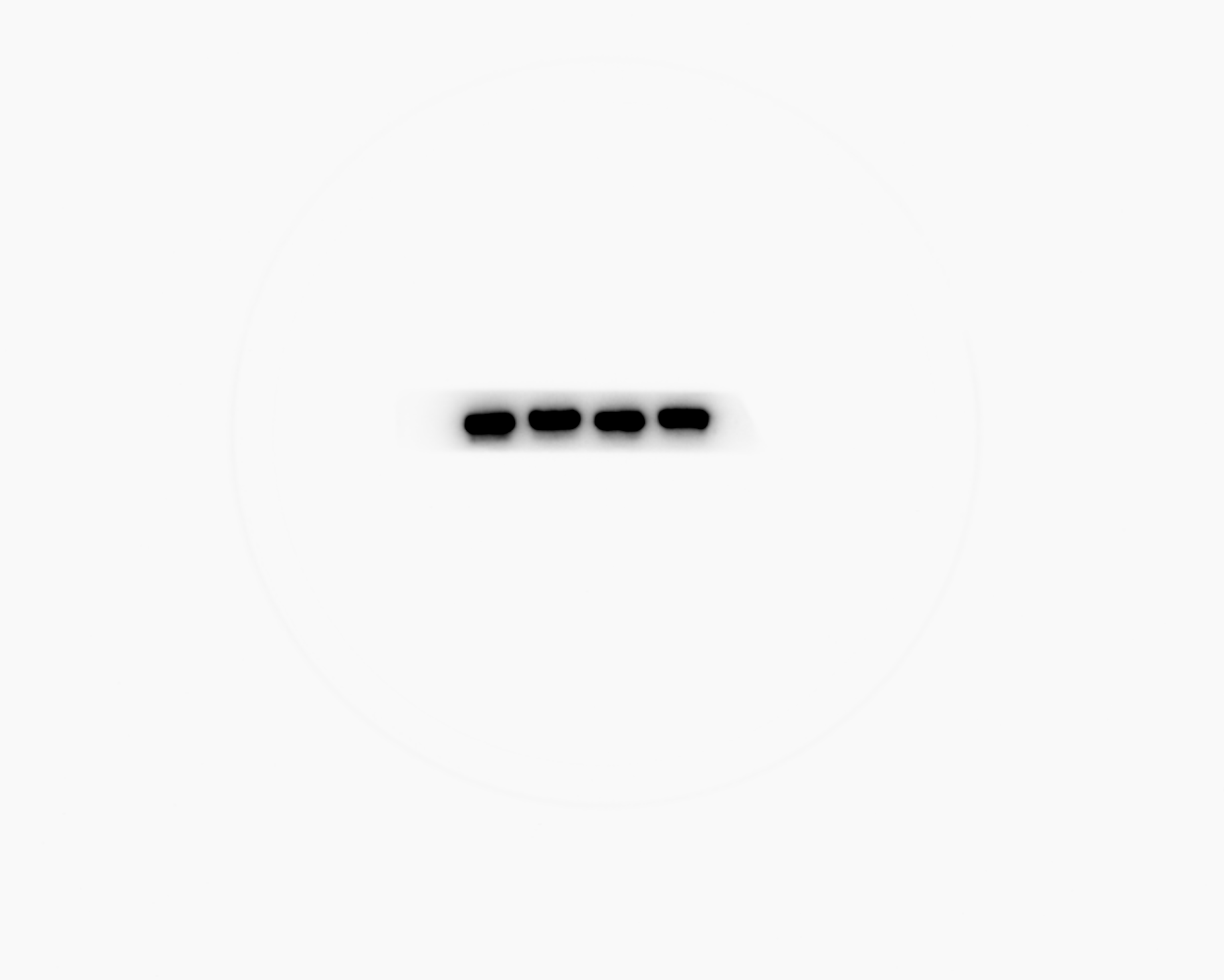

Supplement: Supplementary file 1 [file Presentation_1.zip › original bands for frontiers in pharmacology/GAPDH original bands/GAPDH-3.tif]

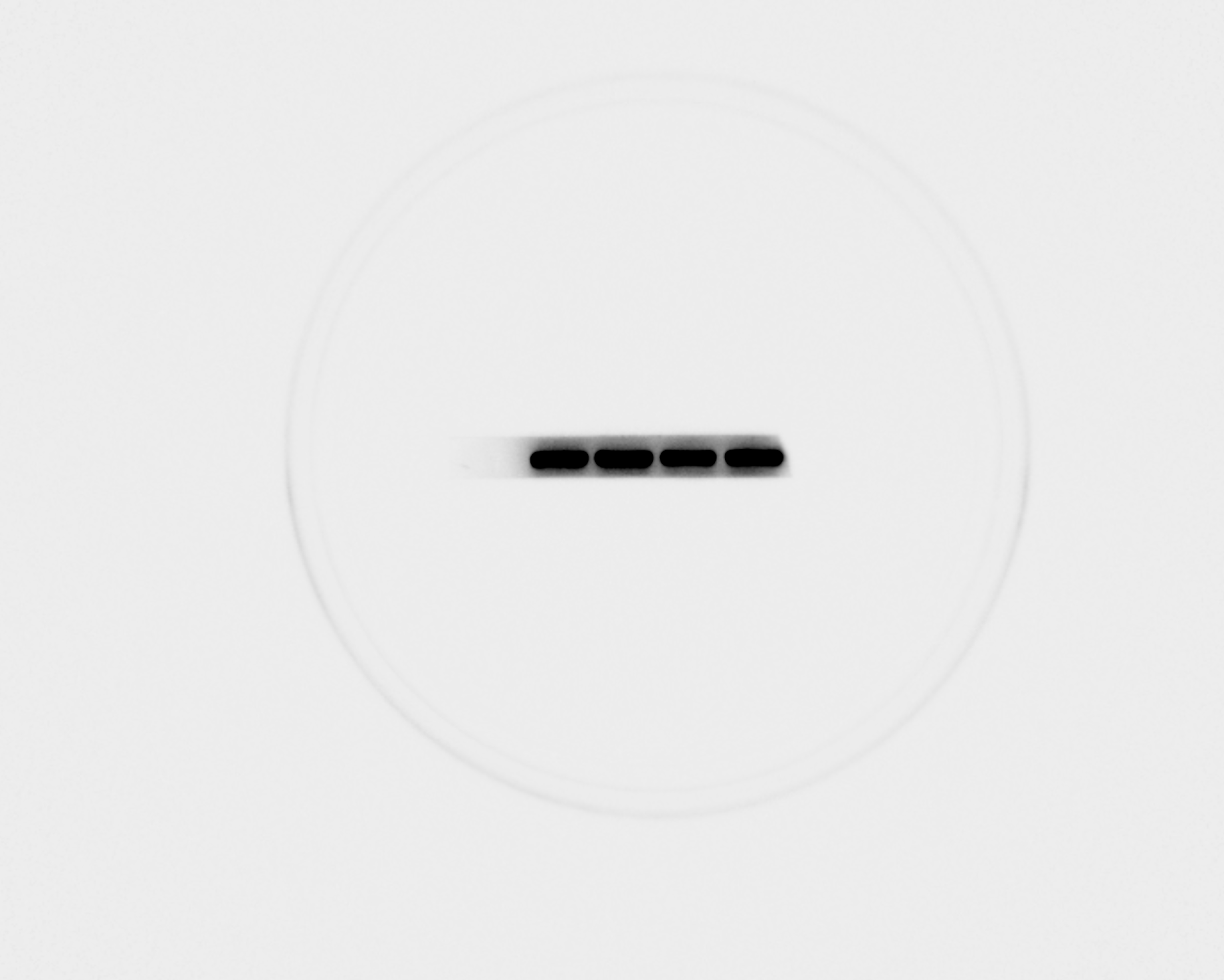

Supplement: Supplementary file 1 [file Presentation_1.zip › original bands for frontiers in pharmacology/GAPDH original bands/GAPDH-4 represent.tif]

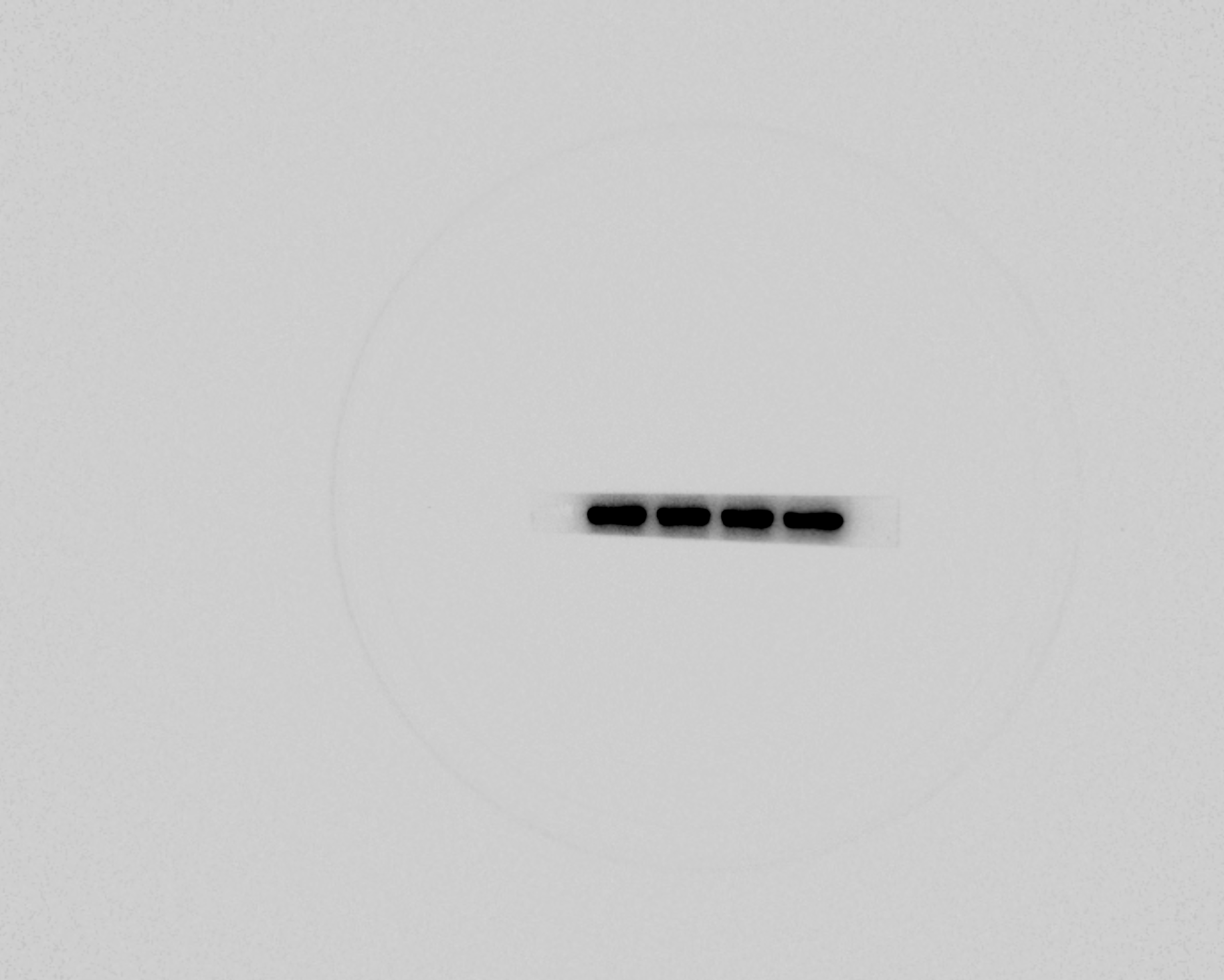

Supplement: Supplementary file 1 [file Presentation_1.zip › original bands for frontiers in pharmacology/GAPDH original bands/GAPDH-5 represent.tif]

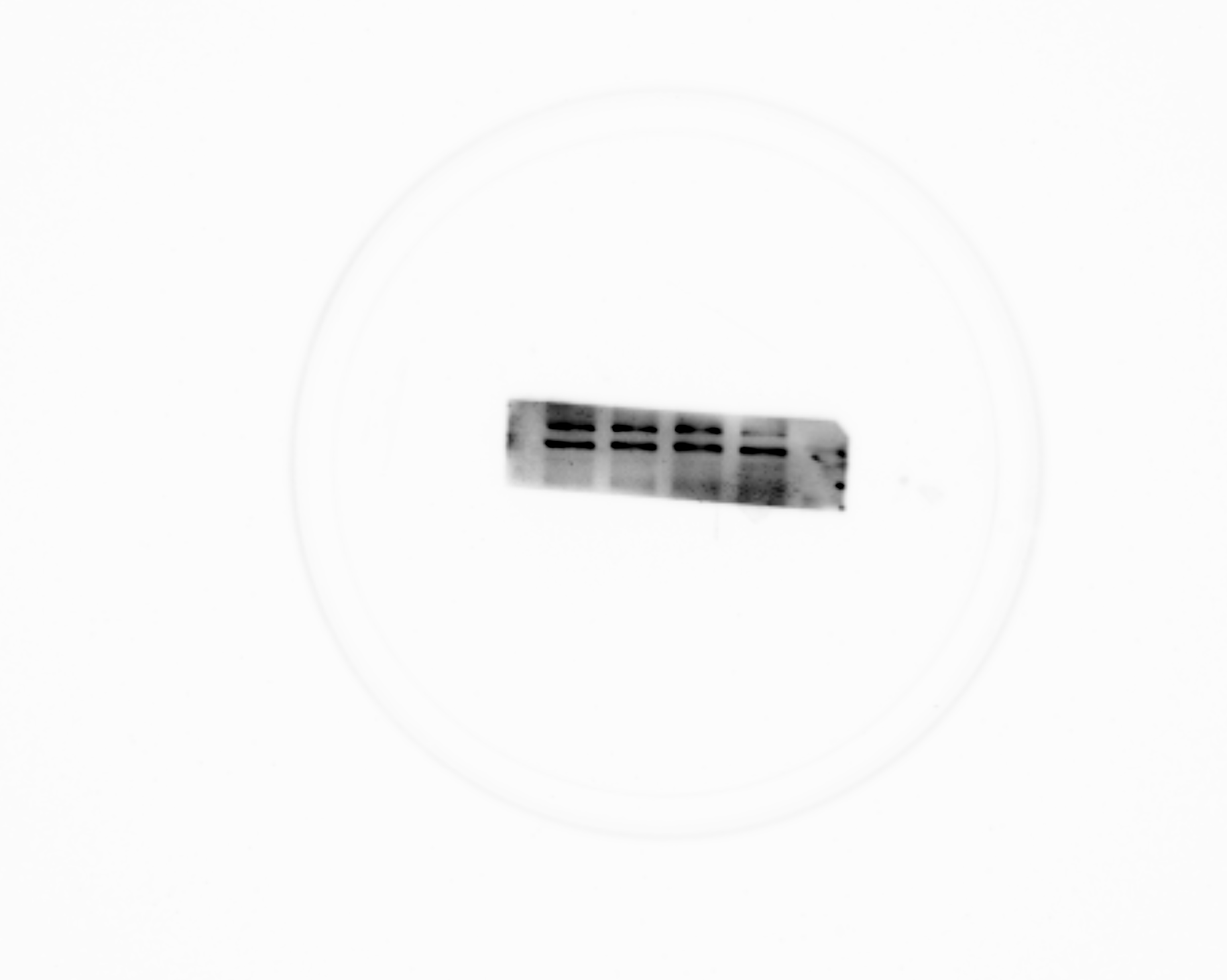

Supplement: Supplementary file 1 [file Presentation_1.zip › original bands for frontiers in pharmacology/p38 original bands/p38-1.tif]

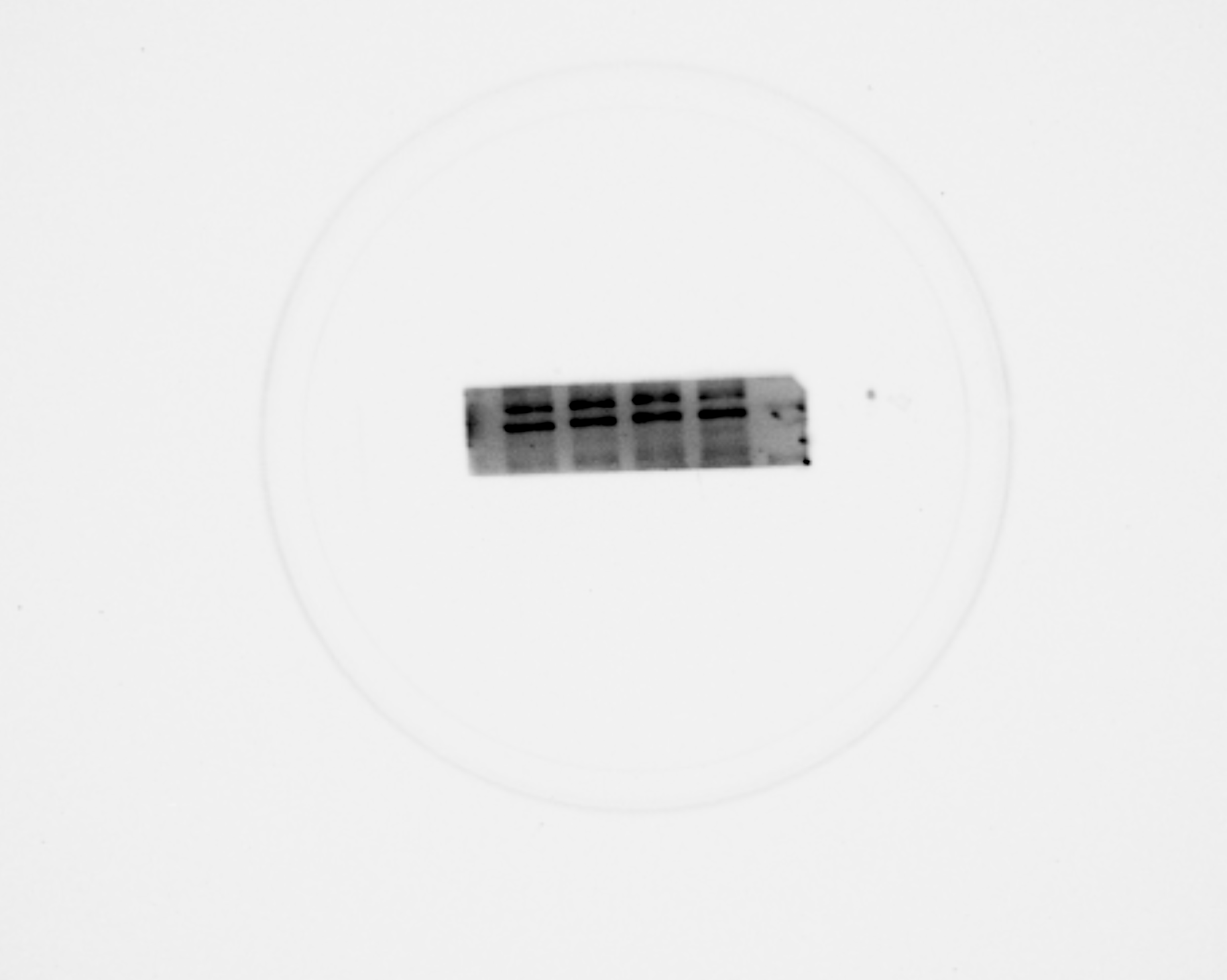

Supplement: Supplementary file 1 [file Presentation_1.zip › original bands for frontiers in pharmacology/p38 original bands/p38-2.tif]

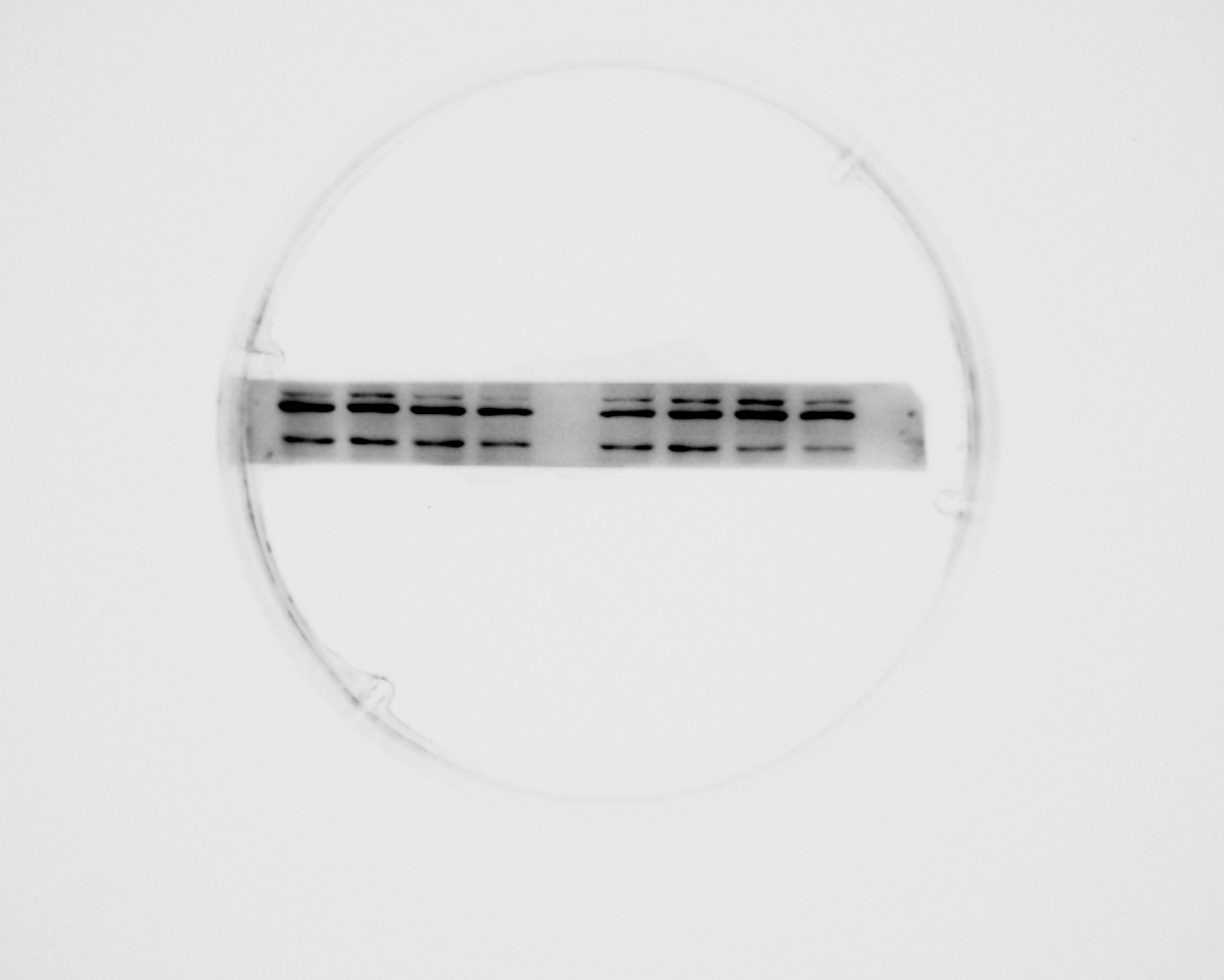

Supplement: Supplementary file 1 [file Presentation_1.zip › original bands for frontiers in pharmacology/p38 original bands/p38-3.tif]

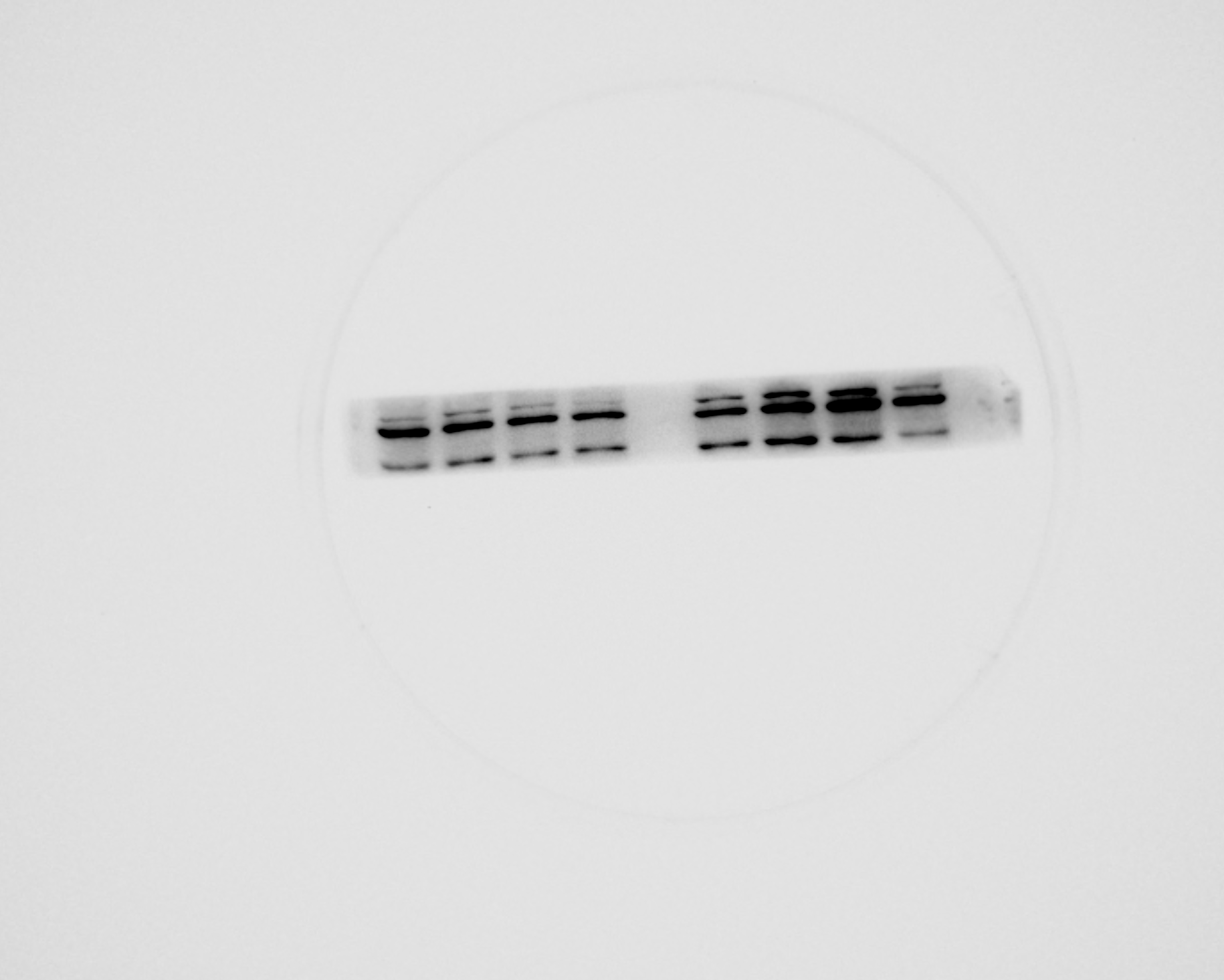

Supplement: Supplementary file 1 [file Presentation_1.zip › original bands for frontiers in pharmacology/p38 original bands/p38-4 represent.tif]

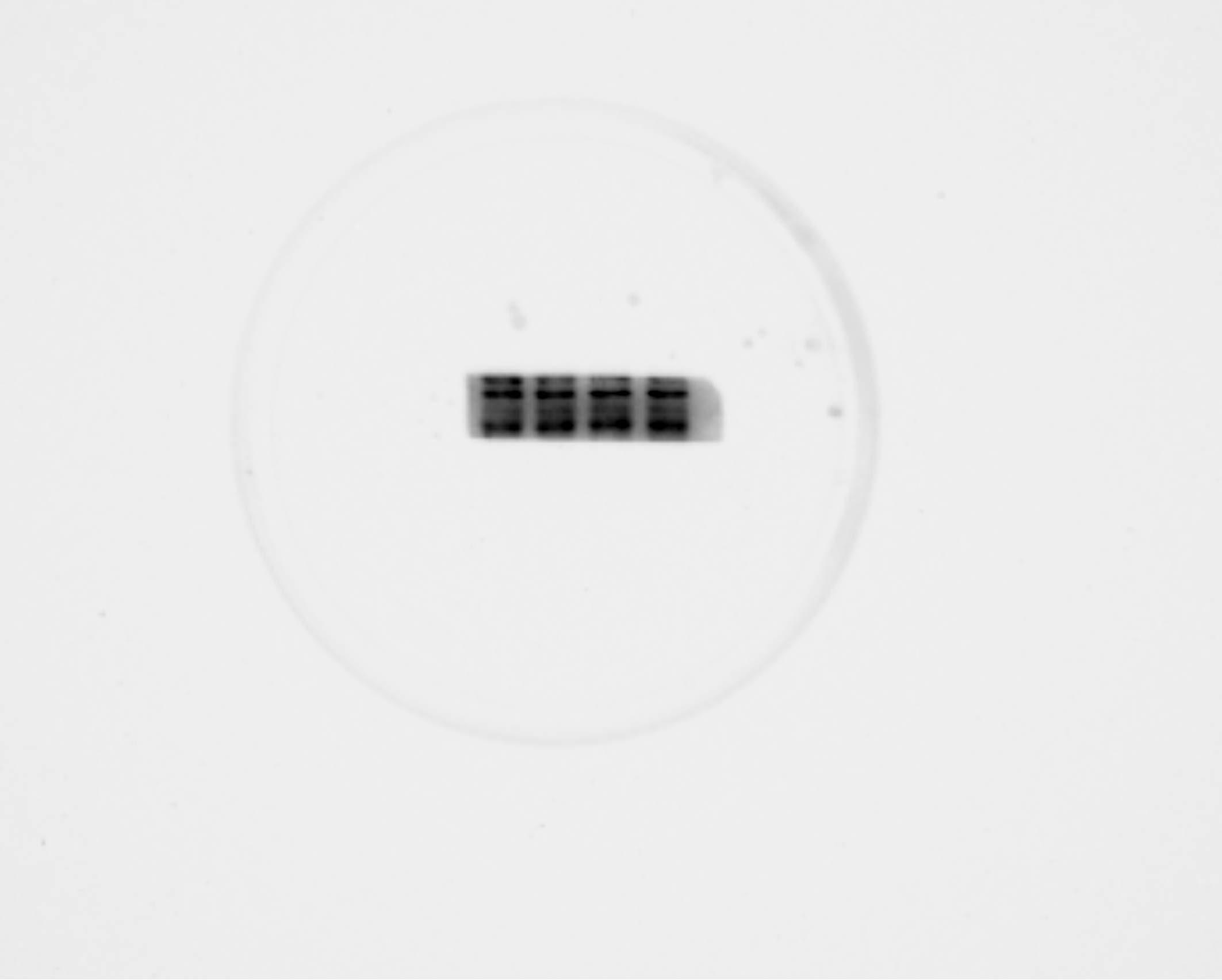

Supplement: Supplementary file 1 [file Presentation_1.zip › original bands for frontiers in pharmacology/p38 original bands/p38-5.tif]

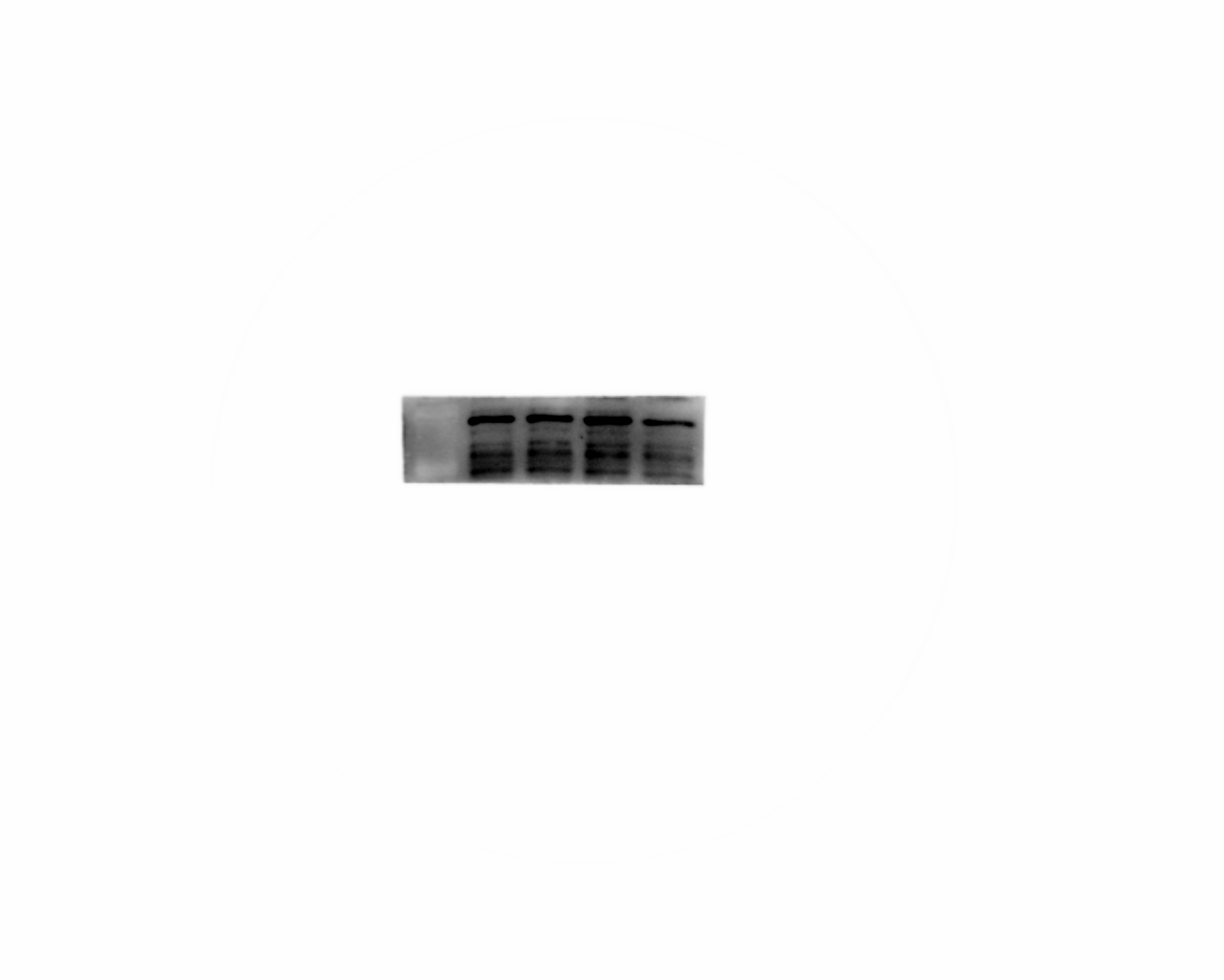

Supplement: Supplementary file 1 [file Presentation_1.zip › original bands for frontiers in pharmacology/p-p38 original bands/p-p38-1 represent.tif]

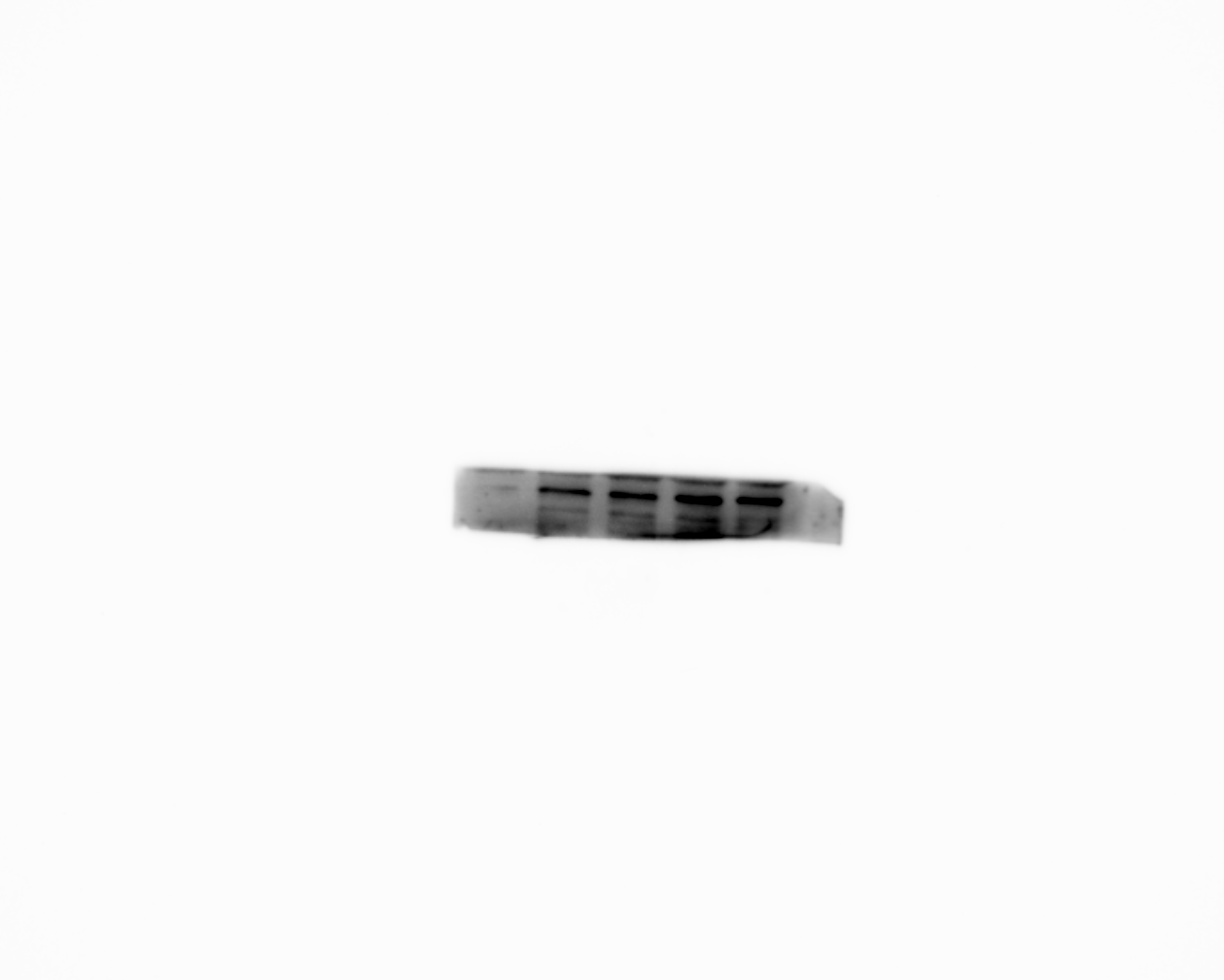

Supplement: Supplementary file 1 [file Presentation_1.zip › original bands for frontiers in pharmacology/p-p38 original bands/p-p38-2.tif]

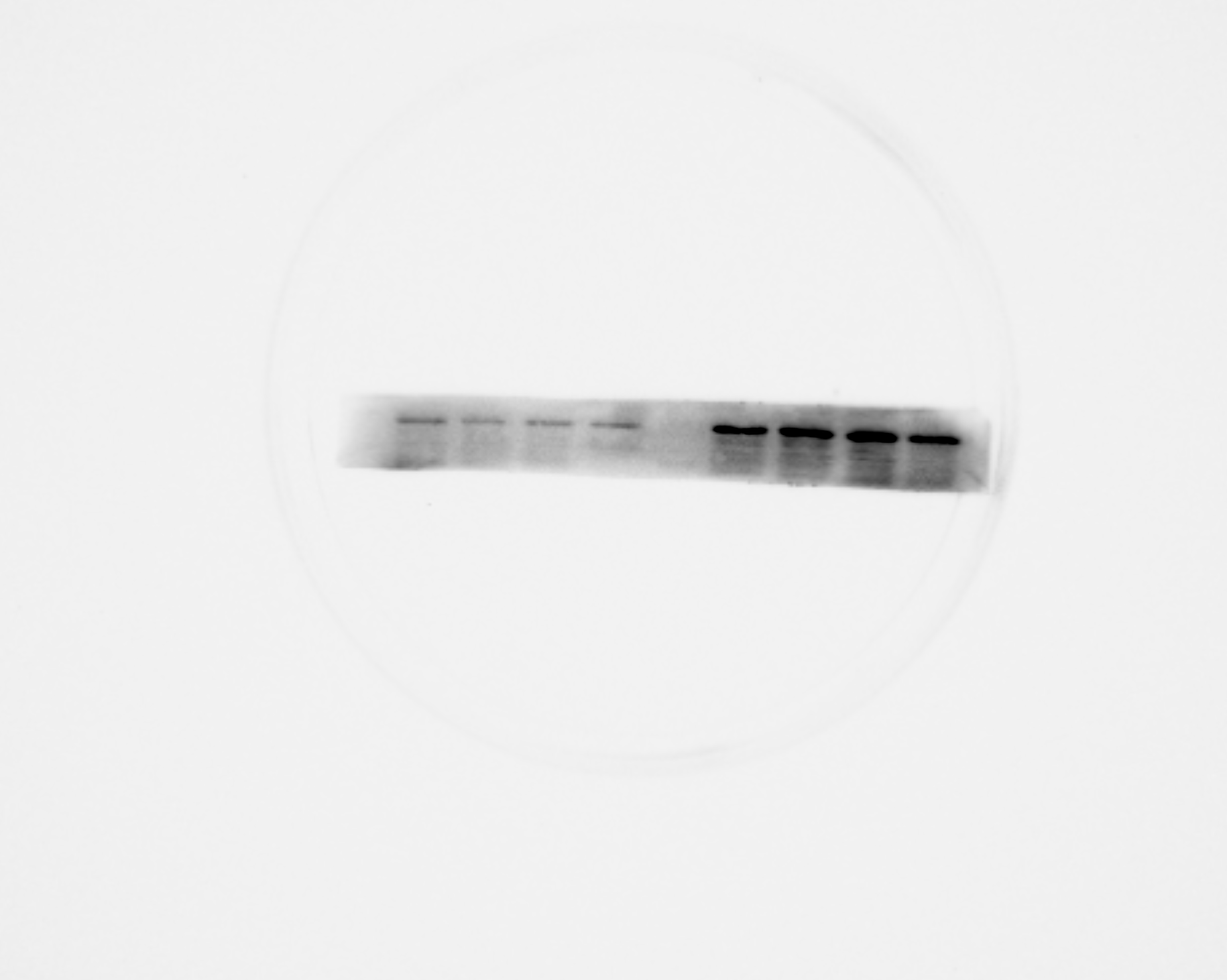

Supplement: Supplementary file 1 [file Presentation_1.zip › original bands for frontiers in pharmacology/p-p38 original bands/p-p38-3.tif]

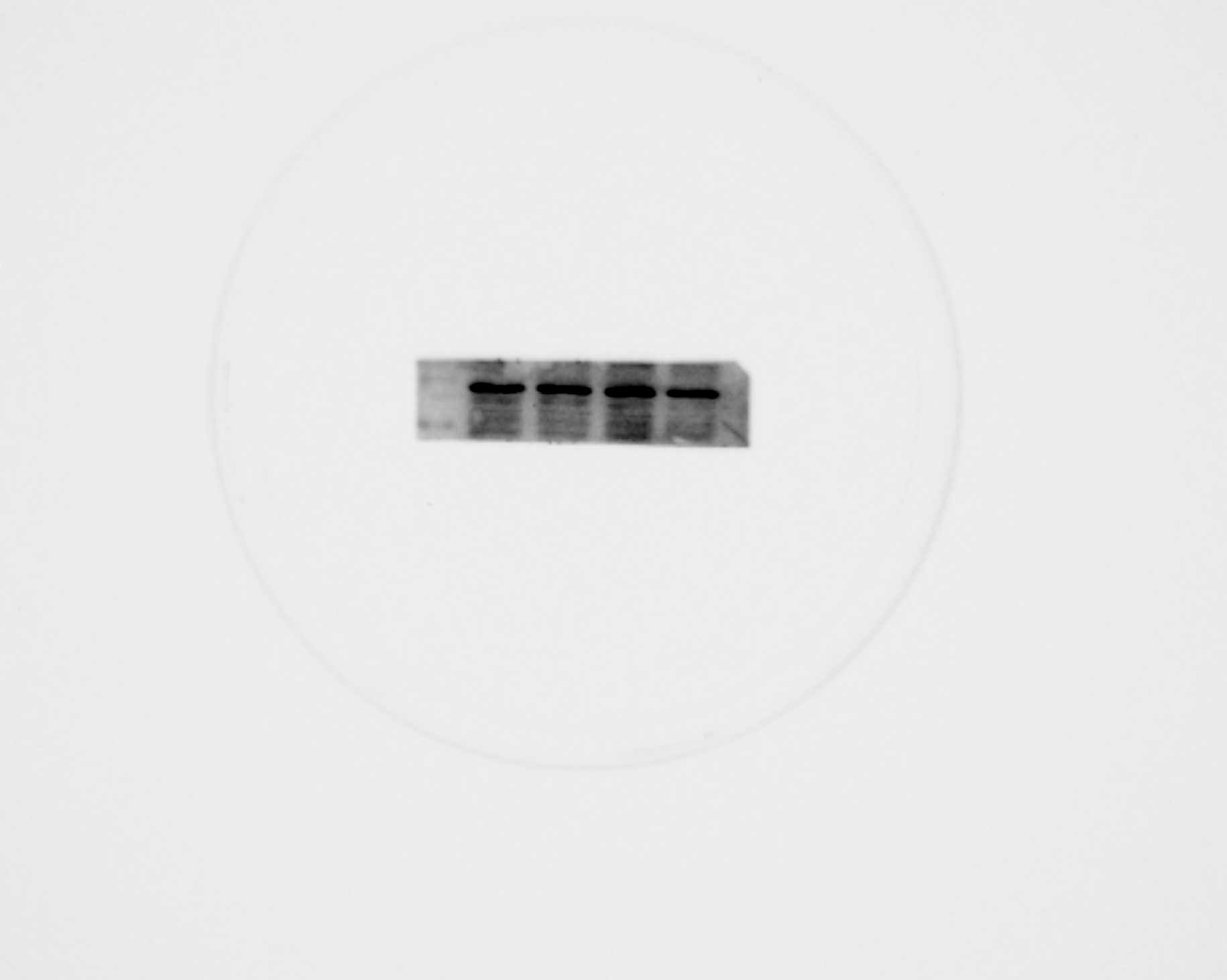

Supplement: Supplementary file 1 [file Presentation_1.zip › original bands for frontiers in pharmacology/p-p38 original bands/p-p38-4.tif]

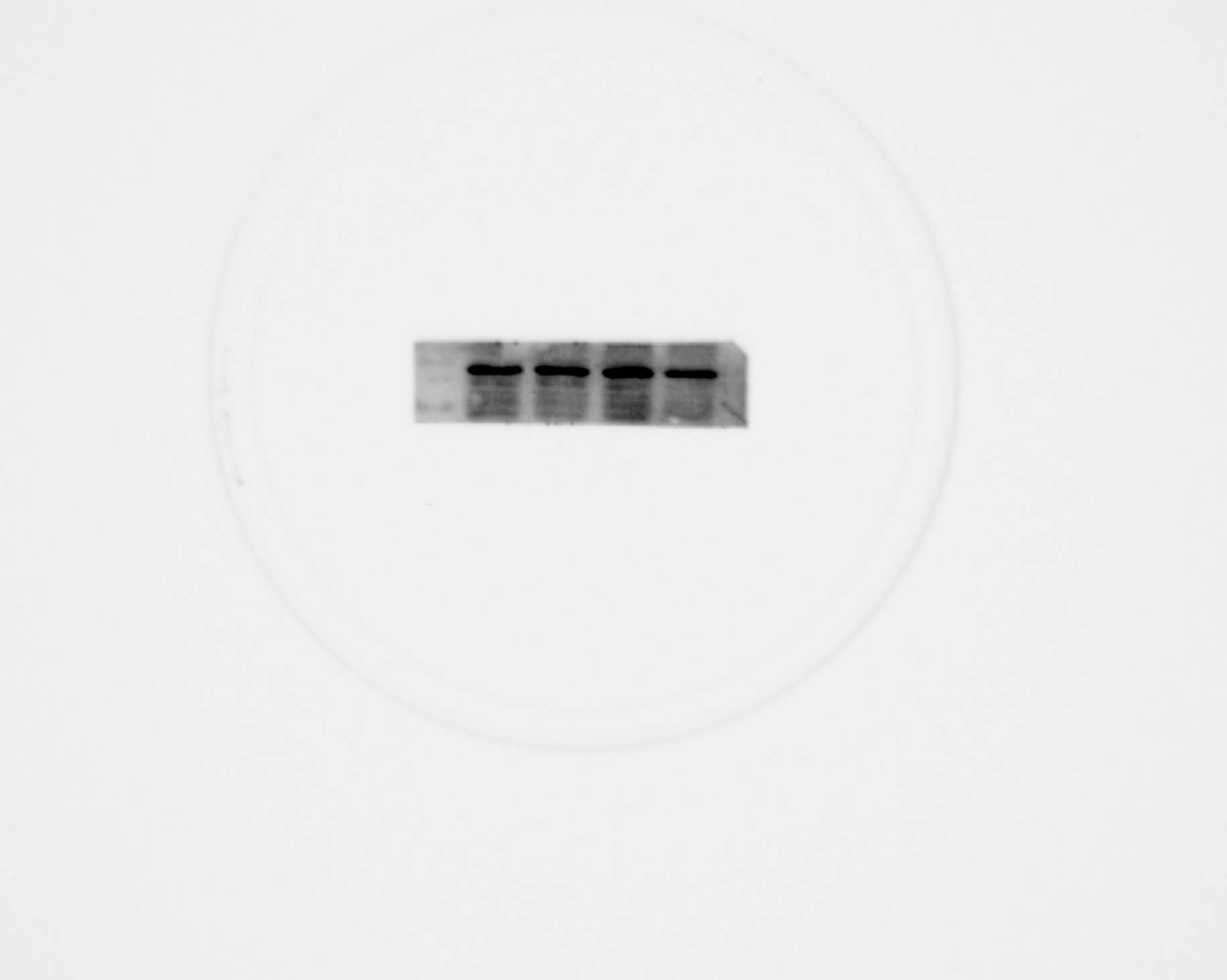

Supplement: Supplementary file 1 [file Presentation_1.zip › original bands for frontiers in pharmacology/p-p38 original bands/p-p38-5.tif]

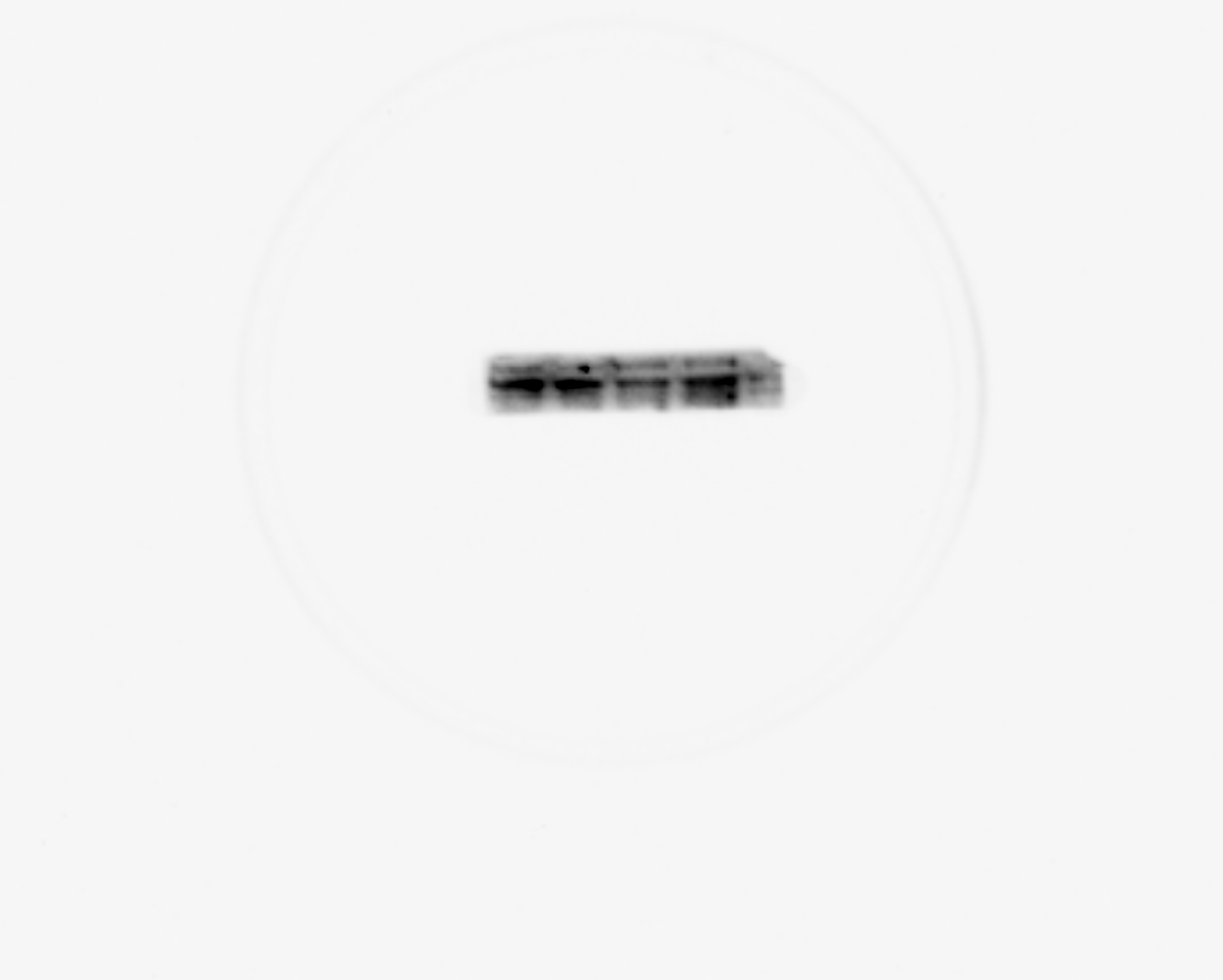

Supplement: Supplementary file 1 [file Presentation_1.zip › original bands for frontiers in pharmacology/Sirt3 original bands/Sirt 3-1.tif]

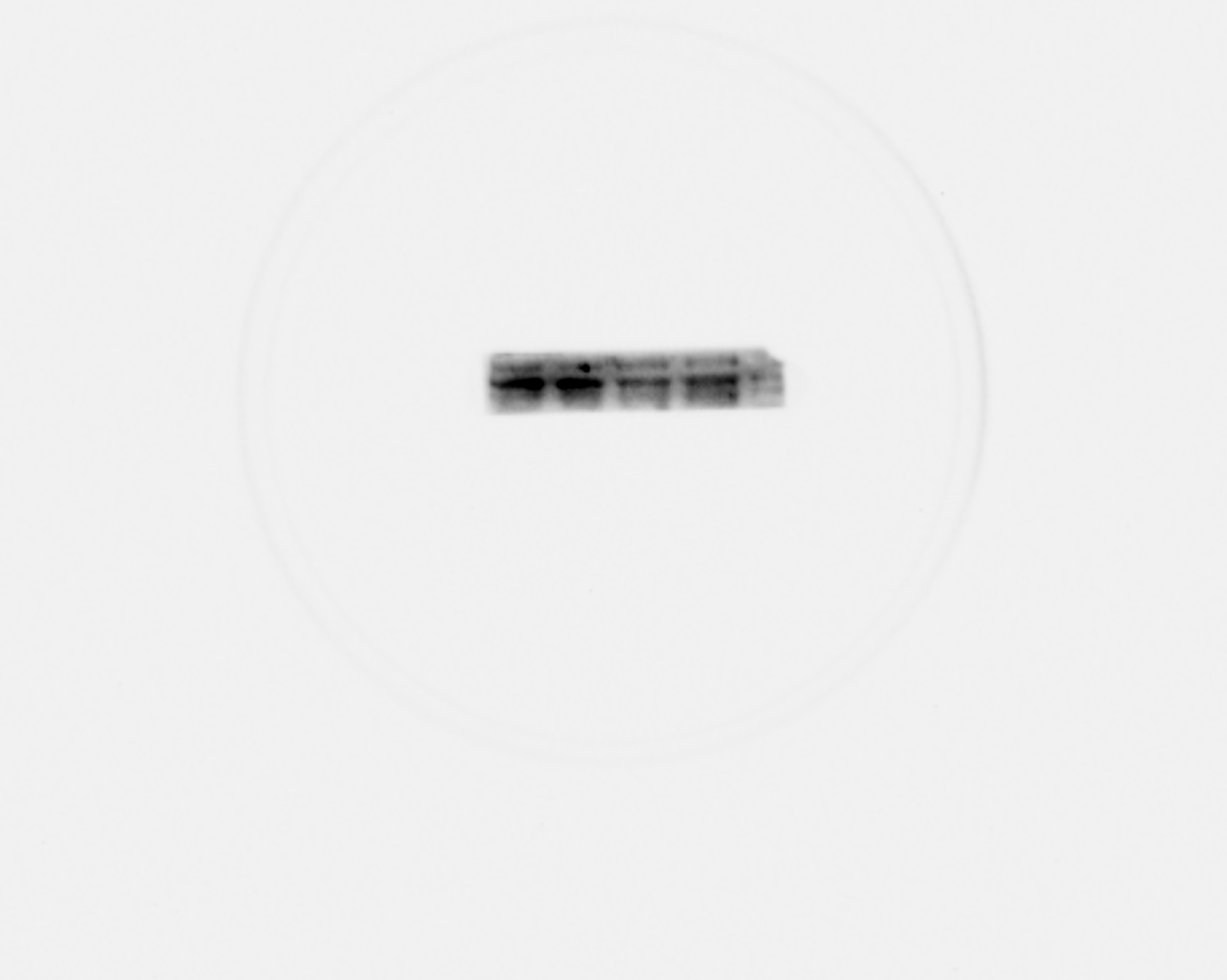

Supplement: Supplementary file 1 [file Presentation_1.zip › original bands for frontiers in pharmacology/Sirt3 original bands/Sirt 3-2.tif]

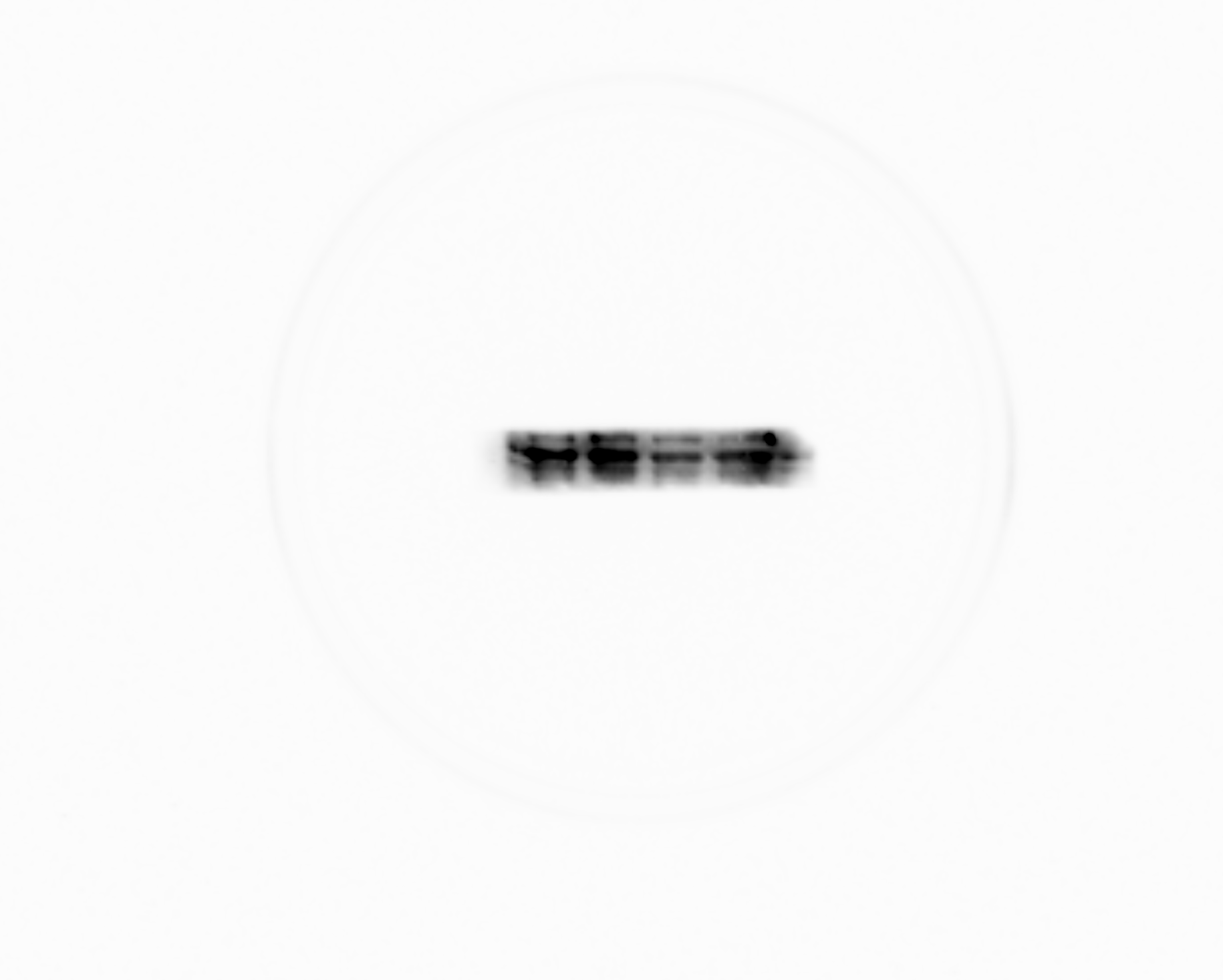

Supplement: Supplementary file 1 [file Presentation_1.zip › original bands for frontiers in pharmacology/Sirt3 original bands/Sirt 3-3.tif]

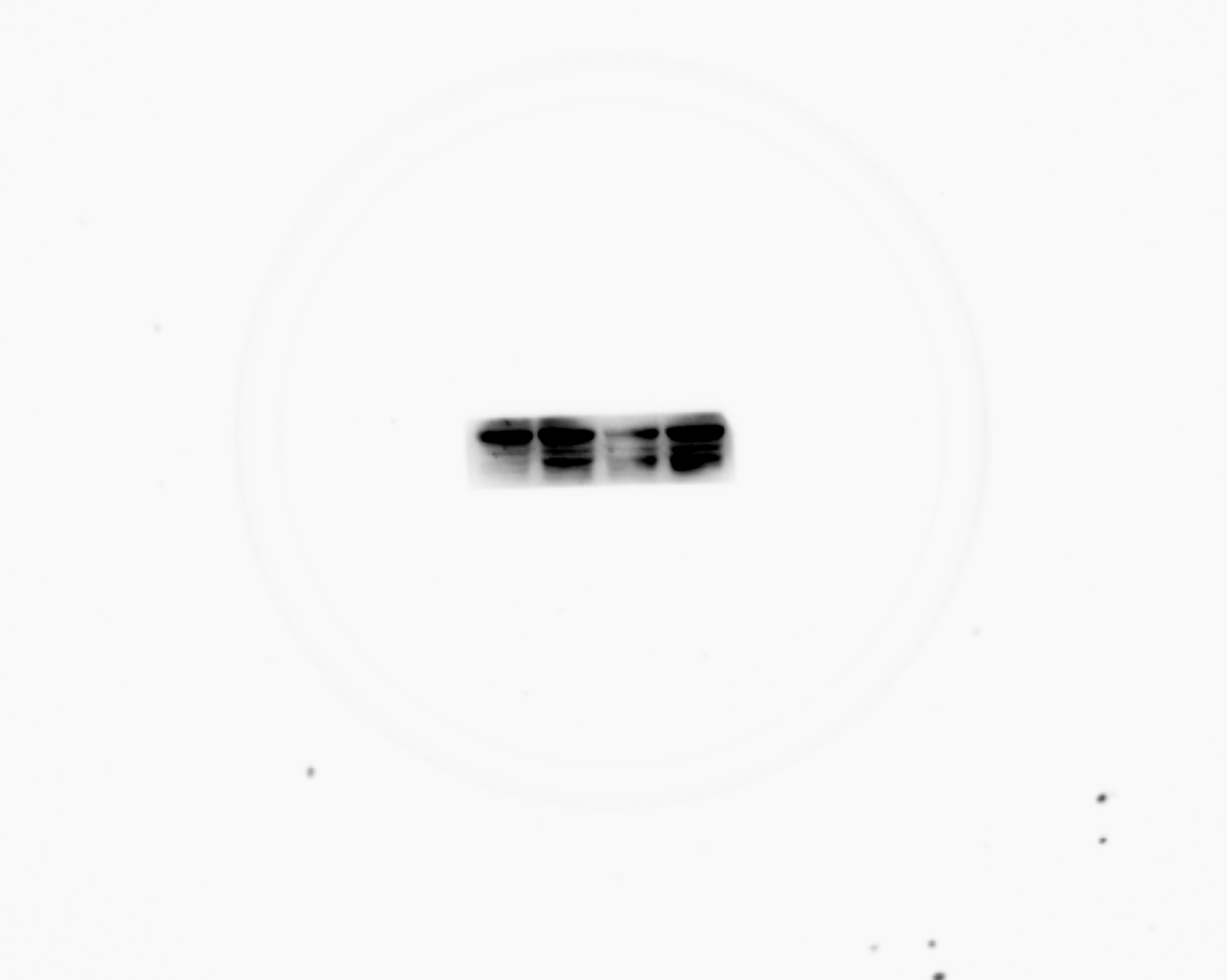

Supplement: Supplementary file 1 [file Presentation_1.zip › original bands for frontiers in pharmacology/Sirt3 original bands/Sirt 3-4.tif]

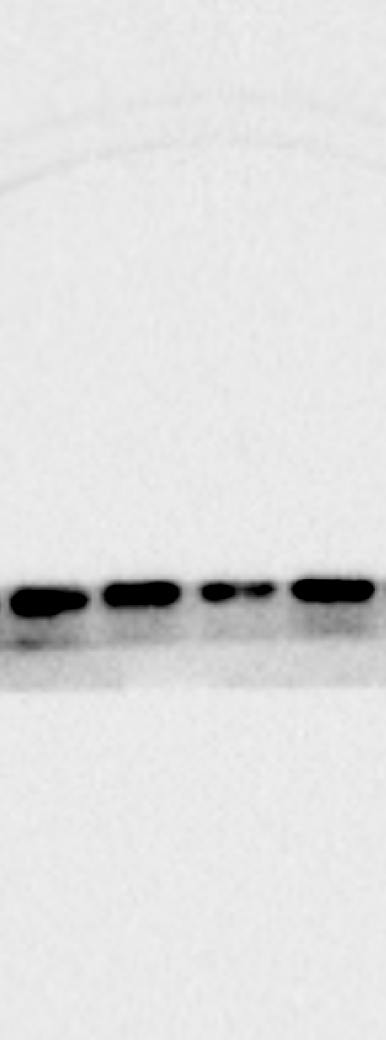

Supplement: Supplementary file 1 [file Presentation_1.zip › original bands for frontiers in pharmacology/Sirt3 original bands/Sirt 3-5 represent.tif]
